# Solutions: how adaptive changes in cellular fluids enable marine life to cope with abiotic stressors

**DOI:** 10.1007/s42995-022-00140-3

**Published:** 2022-08-16

**Authors:** George N. Somero

**Affiliations:** Department of Biology, Hopkins Marine Station, Stanford University, Pacific Grove, CA 93950 USA

**Keywords:** Adaptation, Crowding, Extremophiles, Hydrostatic pressure, Osmolytes, Temperature

## Abstract

The seas confront organisms with a suite of abiotic stressors that pose challenges for physiological activity. Variations in temperature, hydrostatic pressure, and salinity have potential to disrupt structures, and functions of all molecular systems on which life depends. During evolution, sequences of nucleic acids and proteins are adaptively modified to “fit” these macromolecules for function under the particular abiotic conditions of the habitat. Complementing these macromolecular adaptations are alterations in compositions of solutions that bathe macromolecules and affect stabilities of their higher order structures. A primary result of these “micromolecular” adaptations is preservation of optimal balances between conformational rigidity and flexibility of macromolecules. Micromolecular adaptations involve several families of organic osmolytes, with varying effects on macromolecular stability. A given type of osmolyte generally has similar effects on DNA, RNA, proteins and membranes; thus, adaptive regulation of cellular osmolyte pools has a global effect on macromolecules. These effects are mediated largely through influences of osmolytes and macromolecules on water structure and activity. Acclimatory micromolecular responses are often critical in enabling organisms to cope with environmental changes during their lifetimes, for example, during vertical migration in the water column. A species’ breadth of environmental tolerance may depend on how effectively it can vary the osmolyte composition of its cellular fluids in the face of stress. Micromolecular adaptations remain an under-appreciated aspect of evolution and acclimatization. Further study can lead to a better understanding of determinants of environmental tolerance ranges and to biotechnological advances in designing improved stabilizers for biological materials.

## Introduction

The marine environment constitutes greater than 99% of the “living space” (biosphere) of our planet and within this vast volume of water organisms are confronted with a diverse array of challenges by abiotic factors (Torres and Bailey 2022). Temperatures of marine habitats range from slightly below 0 °C in high-latitude waters like the Southern Ocean to temperatures in excess of 100 °C at deep-sea hydrothermal vents. Because most marine species are ectothermic, their cellular temperatures generally closely match those of their habitats. Hyperthermophilic archaea in deep-sea vent waters may thrive at temperatures of ~ 122 °C (Clarke 2014; Kashefi and Lovley 2003), and animal life at vent sites may tolerate exposures to temperatures near 50 °C (Girguis and Lee 2006). The hottest marine animals may be small intertidal invertebrates found at low latitudes that must cope with large increases in temperature during low tides on hot clear days. Certain snails, e.g., the periwinkle *Echinolittorina malaccana*, have been recorded to have body temperatures near 55–57 °C (Dong et al. 2018; Liao et al. 2019; Marshall et al. 2011). Marine species differ greatly in the ranges of body temperatures they encounter, as well as in the absolute temperatures they face. Stenothermal polar species and many deep-sea organisms live at near-constant temperatures, whereas eurythermal intertidal species may experience large changes in body temperatures during the tidal cycle and over the course of the seasons.

The seas also present organisms with a wide range of hydrostatic pressures: pressure increases by 1 atmosphere (1 atm = 1 bar = 0.101 MPa) for each 10 m increase in depth. Thus, in the deepest trenches, pressures exceed 1100 atms (111 MPa). Furthermore, many pelagic species undergo vertical migrations on a diurnal basis or during development, which exposes them to wide ranges of hydrostatic pressure. In some cases, vertical migration also involves changes in body temperature, thereby exposing organisms to changes in two physical stressors.

Salinity (osmolality) is a third abiotic stressor that poses adaptive challenges to marine species. Although most of the open ocean has a stable salinity, considerable variation in salinity is found in some coastal marine habitats, notably in estuarine regions where seasonal changes in precipitation and run-off from land greatly alter the osmolality of the water. Organisms in rocky intertidal habitats in which the osmolality of shallow pools may change greatly due to evaporation or rainfall, also encounter osmotic stress. Likewise, desiccation of emersed intertidal species during periods of low tide can alter the concentration of the solute pools of biological fluids.

### Upsetting the macromolecular stability-flexibility balance: a basic challenge from abiotic stressors

These three abiotic stressors—temperature, pressure and salinity—pose multiple challenges to marine life due to their widespread influences on the structures and functions of all classes of biochemical systems. In this review, I focus largely on one class of stressor effect that challenges the performance of all types of large molecular systems—proteins, nucleic acids and lipoprotein membranes. The two abiotic stressors that will be of central focus are temperature and hydrostatic pressure, both of which have pervasive effects on large molecular systems. I show that the perturbing effects of these two stressors at the biochemical level often result from their potential to disrupt the fine balance that is needed between stability and flexibility of the higher-order structures of these large molecular systems, which are stabilized largely by non-covalent (weak) chemical bonds like hydrogen bonds, ionic interactions, and hydrophobic effects (Liao et al. 2019, 2021; Somero 2022). Maintaining this delicate balance is essential for sustaining optimal biochemical function. Disruption of higher-order macromolecular structures is a pivotal factor in triggering the energetically costly cellular stress response (CSR), so the stability-flexibility balance in large molecular structures is a critical factor in governing cells’ responses to all categories of stressors and in establishing the costs of using the CSR (Kültz 2020).

The fundamental basis of this requirement for an appropriate balance between stability and flexibility in large molecular systems is the requirement for these dynamic systems to have a specific three-dimensional configuration (conformation) to allow recognition of other molecules, while retaining the ability to alter conformation to perform the required function. This is a wide-ranging requirement that includes assembly of subunits of multimeric proteins, formation of nucleic acid-protein complexes, and many other processes in which multi-macromolecule assemblages are formed. A relatively simple illustration of the dynamic balancing act found in macromolecular structure is seen in enzyme-catalyzed reactions. An enzyme must have a specific geometry (stereochemistry) to allow it to recognize and bind the substrates of the reaction it catalyzes. Additionally, however, proteins must be flexible enough to undergo changes in conformation during their activities. Conformational changes in enzymes are essential parts of the catalytic cycle and often are the rate-limiting event in enzyme function. Thus, an overly rigid enzyme may function poorly, even if this rigidity should make it relatively resistant to physical stressors.

In nucleic acids (RNA and DNA), changes in higher order structures are likewise important in conducting numerous key functions. For example, changes in the secondary structures of messenger RNAs (mRNAs) are needed to govern several events during the process of translation (Liao et al. 2021; Mortimer et al. 2014). Overly rigid secondary structures may block initiation of mRNA translation, reduce rates of translation, impede splicing at intron–exon junctions, and reduce the ability of the mRNA to sense changes in its physical and chemical environment that may play important roles in regulating gene expression. For example, temperature-driven changes in secondary structures of certain mRNAs may enable them to serve as cellular thermometers that regulate initiation of translation (Somero 2018). RNA thermometers must “melt” at the right temperatures if their regulatory role is to be achieved; too rigid a secondary structure may preclude initiation of translation when the need for the gene product, e.g., a protein of the CSR, arises at higher temperatures. Overly labile RNA structures also can have negative effects on RNA function if this high lability switches the thermometer on at too low a temperature. Pressure effects on RNA and DNA also lead to a need for retention or restoration of the stability–flexibility balance in the face of this abiotic stressor (Arns et al. 2019; Patra et al. 2018).

Analogous balancing act phenomena pertain in the case of cellular membranes, where three critical balances must be present: (1) retention of the appropriate liquid–crystalline state (fluidity) of the bilayer, (2) control of transitions between the liquid–crystalline state and the gel phase, and (3) the appropriate capacity to generate non-lamellar structures, for example, during endo- and exo-cytosis (Hazel 1995; Somero et al. 2017). The static order of the lipid bilayer influences the activity and mobility of membrane proteins, which commonly intercalate into the bilayer and may span it in many cases, e.g., ion transport proteins. Direct links between the biophysical state of membranes and cellular metabolism have been discovered. For instance, the viscosity of lipids may govern the activity of the electron transport system (ETS) by influencing the rates of diffusion-controlled reactions between ETS enzymes and electron carriers (Budin et al. 2018). Formation and function of lipid rafts may also depend on membrane biophysical properties. The transition from a liquid–crystalline state to a gel phase has significant effects on the barrier functions of membranes (Hazel 1995). The capacity to generate non-lamellar structures, for example, during release of neurotransmitters into the synaptic cleft, depends on the capacity of the bilayer to undergo phase changes from lamellar to hexagonal geometries (Hazel 1995).

These important characteristics of membranes’ biophysical and structural properties are highly sensitive to changes in temperature and hydrostatic pressure (Cossins and Macdonald 1989). In some circumstances, notably in the deep sea, synergistic effects occur between these two abiotic stressors, creating especially significant challenges for the retention of an optimal stability-flexibility balance to support membranes’ diverse functions (Cossins and Macdonald 1989; Manisegaran et al. 2019).

In summary, all of the cell’s large molecular systems must strike a balance between flexibility and stability of structure if physiological functions are to occur optimally. This requirement spans all taxa and thus we would anticipate that major evolutionary selection for optimal states of stability would be manifested in all forms of life. Likewise, for marine species that encounter changes in temperature, pressure, and salinity during their lifetimes, we would anticipate important acclimatization changes to ensure sustained optimality of function.

### Achieving the stability-flexibility balance: roles of intrinsic and extrinsic factors

This physiologically important balance between stability and flexibility of structure in large molecular systems is achieved in two principal manners (Somero 2022). First, during evolution, the abiotic conditions that an organism faces lead to genetically based adaptations in the conformational stabilities of proteins (Dong et al. 2018, 2022; Liao et al. 2019; Somero 1995, 2022) and certain types of nucleic acids, notably messenger RNAs (mRNAs) (Liao et al. 2021). In general, the higher the adaptation temperature, the more intrinsically rigid are the conformations of nucleic acids (Liao et al. 2021) and proteins (Somero et al. 2017). I term these *intrinsic* adaptations to denote that they are encoded in the genome of the organism (Somero 2003). Often, only minor differences in protein or RNA sequence are required to achieve these adaptive changes in stability (Dong and Somero 2008; Fields et al. 2006, 2015; Liao et al. 2021; Lockwood and Somero 2012). Differences in lipid compositions that derive from adaptive modifications of the biochemical pathways that promote lipid biosynthesis might also be grouped into the category of intrinsic adaptations, because these evolved differences in lipid composition may achieve appropriate adjustments in the stability/flexibility balance in lipid-containing systems like membranes. The structural stability/flexibility balance requirement extends as well to depot lipids, which must have an optimal structural state to allow their utilization (for review: Somero et al. 2017).

Complementing these intrinsic, sequence-based adaptations in macromolecular structure are alterations in the chemical compositions—the “micromolecular” contents—of biological solutions that bathe macromolecules and influence their stabilities and functions (Yancey 2005, 2020). I refer to these as *extrinsic* adaptations to emphasize the distinction between adaptive differences in structure and chemical composition that determine intrinsic conformational stability of nucleic acids and proteins and biophysical properties of lipids and the solution-determined effects of micromolecules that facilitate retention of these evolved differences under different environmental conditions. Within the generic category “micromolecules,” I group the small organic molecules and inorganic ions that comprise the greater share of the cell’s osmotic pool and protons, which play pivotal roles in pH-dependent processes. I shall not discuss pH relationships in this review, albeit they are obviously of key importance in physiological systems (for review, see Somero et al. 2017)). The small organic molecules and inorganic ions I focus on are commonly referred to as *osmolytes*, to denote their central roles in determining the osmolality of biological solutions. I will employ this conventional terminology in this review, although as we will see, the roles of these osmolytes extend far beyond just the regulation of osmolality. Most importantly, this analysis will show how critical adaptive changes in osmolyte systems are in facilitating conservation of the stability-flexibility balance of macromolecular structure to allow life to thrive over the wide ranges of abiotic factors found in the marine realm. 

The study of adaptive macromolecular-micromolecular interactions has multiple benefits. First, we can learn important lessons about biochemical evolution from investigating the inventions used by marine life to cope with the challenges posed by the abiotic factors they face. Importantly, we learn that meeting this challenge requires a team effort that involves both the large and small molecular components of cells. Neither intrinsic nor extrinsic adaptations on their own may be able to solve the challenges posed by abiotic stressors. Second, by learning how evolution has fostered developments of solutions with favorable effects on large molecular systems like proteins, nucleic acids and membranes, we may gain insight into biotechnological means for stabilizing many labile study systems. Third, our understanding of biological solutions should have important influences on how we design our experiments, notably in vitro studies in which solution properties have a strong influence on what we observe. At several points, I offer caveats about the importance of creating a realistic micromolecular environment for discovering the true in vivo properties of the large molecular systems being studied.

### Water: a medium and a means of mediation of solute effects

Water is the most abundant chemical in living systems, and it affects almost every aspect of biochemical structure and function. In its role as a powerful solvent, cellular water provides the dissolved materials needed by enzymes and other macromolecules to perform their diverse functions. In this role, water may seem to be merely an inert container of the biochemical systems that are most pivotal to life. However, water’s roles are much more extensive than this. The water that bathes macromolecules and membranes helps to determine many of their key properties, notably their three-dimensional shapes and conformational stabilities, which depend strongly on the hydrophobic effect—the withdrawal of non-polar entities like the large amino acid sidechains of valine and isoleucine, from contact with water. The bilayer structure of cellular membranes also reflects hydrophobic behavior, in this case by acyl chains of membrane phospholipids. In turn, both macromolecules and osmolytes have profound effects on the structure of water. These water-macromolecule-osmolyte interactions lead to marked heterogeneity in water structure in biological solutions. For example, osmolytes—both small organic molecules and inorganic ions—can be either water-structure-makers or water-structure-breakers. The water-organizing characteristics of an osmolyte play a central role in establishing the stabilizing or destabilizing effect of the solute on macromolecules (Auton et al. 2011; Bolen 2004; Bolen and Rose 2008; Hishida et al. 2022). A strong positive correlation is found between an osmolyte’s water structuring ability, as indexed by its ability to reduce the mobility of water molecules, and its capacity to stabilize proteins (Hishida et al. 2022). The structure of the water that coats macromolecules differs from that of so-called bulk water. The abilities of osmolytes to interact closely with the surface of a macromolecule are determined by the structured water near the macromolecular surface as well as by the structure of the water surrounding the osmolyte itself. Thus, water is not only the medium in which biochemical activity takes place, but surface- and solute-induced changes in its structure provide the means by which the effects of micromolecules on macromolecules are mediated.

The study of water-solute interactions involving osmolytes of marine species has two principal benefits. First, these investigations are providing insights into the adaptive processes in these organisms that allow them to succeed in widely different environmental conditions. Secondly, this work is shedding new light on the fundamental biophysics of aqueous biochemical systems. Thus, the studies I describe below not only offer marine biologists important new information on how marine life adapts to the abiotic stressors of the sea, but these investigations also are teaching physical biochemists critical things about the physics of water-solute interactions and, for the technologically minded, are suggesting new strategies for developing solutions that aid in the stabilization and preservation of biological materials.

## Osmotic adaptation: basic patterns of osmolyte distribution

Osmotic composition and osmolality vary widely among the diverse taxa found in the oceans. In multicellular organisms that have extracellular fluids as well as intracellular solutions, there is invariably a large difference in the compositions, but not the total osmolalities of the different fluid compartments. The focus of the analysis that follows will be almost exclusively on *intracellular* osmolytes because comparisons will span both multicellular and single-cell organisms found across all domains of life: Archaea, Bacteria, and Eukarya. The qualitative and quantitative properties of intracellular osmolyte pools have been characterized in numerous marine species representing all three domains of life (for review, see Yancey 2005; Yancey et al. 1982). Here, I provide only a brief overview of the most important characteristics of osmolyte systems, to set the stage for analyzing the selective advantages of different types of osmolytes in generating a cellular solution that is optimal for macromolecular structure and function.

### Intracellular inorganic ion concentrations are widely conserved across marine taxa

Several characteristics of osmolyte systems bear emphasis for this purpose. First, the types and concentrations of inorganic ions found in intracellular fluids exhibit striking consistency across diverse taxa (Yancey et al. 1982). Within the intracellular fluids, potassium ion rather than sodium ion is the dominant inorganic cation in all species so studied. Sodium is the dominant cation in extracellular fluids. Moreover, despite wide variation in total cellular osmolality, K^+^ concentrations span a relatively narrow range across most marine species (~ 150–200 mmol/kg), with extremely halophilic archaea being a notable exception (Yancey et al. 1982). The relative consistency and stability of inorganic ions’ contributions to the osmolality of cellular solutions reflects their strong effects on large molecular systems. High and variable concentrations of inorganic ions can be highly perturbing of macromolecular structure and function, for example by inhibiting enzyme activity (Bowlus and Somero 1979; Yancey et al. 1982), by causing double-strand breaks in DNA (Dmitrieva et al. 2006; Kültz and Chakravarty 2001), by affecting higher order structures of RNA (Arns et al. 2019; Russell et al. 2002), and by upsetting transmembrane ion gradients needed for neural transmission and uptake of small molecules into cells.

### Organic osmolytes are the primary focus of adaptive change

These diverse and widespread perturbing properties of inorganic ions likely account for a second important general observation: during both long-term evolutionary adaptation and phenotypic acclimatization, adjustments to intracellular osmolality have consistently favored altering concentrations of organic osmolytes, not inorganic ions. In contrast to the disruptive effects of changes in concentration of inorganic ions, the organic osmolytes accumulated at variable and sometimes extremely high concentrations are generally non-perturbing or stabilizing of macromolecular structure, at least at physiological concentrations. Thus, these osmolytes have come to be called *compatible* solutes, a terminology introduced by Austen Brown in the 1970s (Brown 1976). However, non-physiologically high concentrations of compatible solutes or their accumulation to high levels in the absence of a stressor that challenges macromolecular structure and function can be maladaptive, as discussed later (see Yancey 2005).

**Fig. 1:**
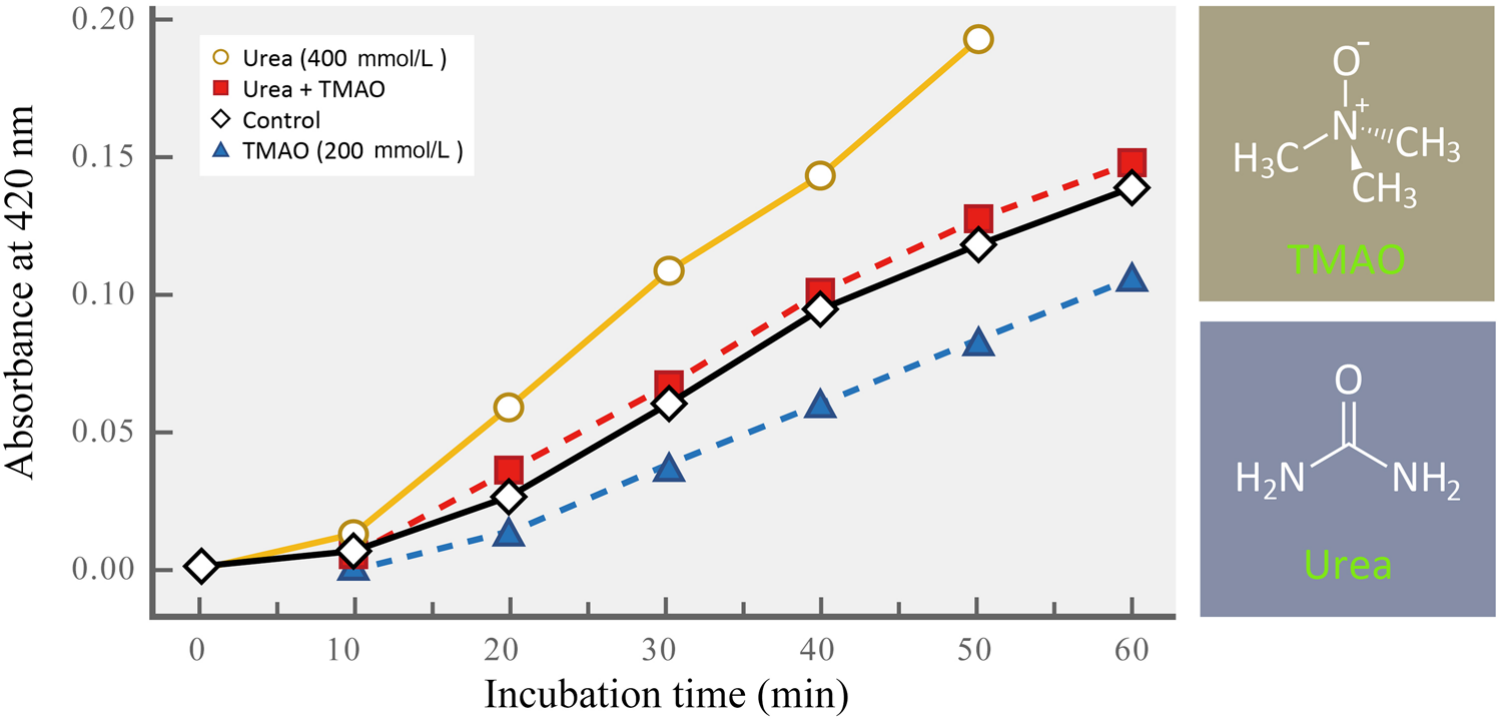
The effects of TMAO and urea on the rate of labeling of sulfhydryl groups of glutamate dehydrogenase by the reagent 4-chloro-7-nitrobenzofurazan (Nbf-Cl). Control mixtures had neither TMAO nor urea. The structures of TMAO and urea are shown to the right of the graph. (Figure redrawn after Yancey and Somero 1979)

The organic osmolytes selected for accumulation at high and/or variable concentrations show strong similarities among widely different taxa (Yancey 2005; Yancey and Siebenaller 2015), albeit extremophiles tolerant of high temperatures and/or high salinities produce unique osmolytes not found in mesophilic species, as discussed later (Santos and da Costa 2002). Regardless of species’ adaptation temperatures, pressures, or salinities, organic osmolytes belong to only a small number of categories: *sugars *and *polyhydric alcohols* (polyols) like trehalose and glycerol, respectively; *amino acids* (e.g., proline and aspartate) and their breakdown products (e.g., taurine); *methylammonium compounds* like trimethylamine-N-oxide (TMAO) (Fig. 1), glycine betaine (GB), and glycerophosphorylcholine (GPC), *methylsulfonium compounds* like β-dimethylsulfoniopropionate, and *urea* (Fig. 1). The choice of osmolytes used by different species may be based on several factors, including ease of obtaining the compounds through de novo synthesis or from dietary sources. However, issues of osmolyte supply are likely of secondary importance in shaping the composition of the osmolyte pool, relative to the needs of an organism for maintaining the optimal level of macromolecular stability under the conditions it faces in its habitat. The diversity of organic osmolytes available for this purpose allows the stabilizing efficacy of the osmolyte pool to be finely tuned to generate conditions favoring an optimal level of molecular stability. As Bolen (2004) points out, there is truly a continuum of solute effects on macromolecules; the variation in osmolyte composition found under different conditions of abiotic stress reflects the effectiveness with which organisms have exploited this raw material.

Two early examples of the differing effects of osmolytes on proteins remain paradigmatic in illustrating the diversity of osmolyte effects and the potential for developing osmolyte mixtures with appropriate stabilizing properties. Figure 1 shows the opposing effects of urea, a strong denaturant of macromolecules, and the structure-stabilizing methylammonium osmolyte trimethylamine-N-oxide (TMAO) on protein stability. Here, the conformational flexibility of the protein is indexed by the rates at which sulfhydryl groups on the enzyme glutamate dehydrogenase (GDH) are labeled by an organic tagging reagent (4-chloro-7-nitrobenzofurazen (Nbf-Cl) in the presence of different combinations of urea and TMAO (Yancey and Somero 1979). Access to the buried sulfhydryl groups is provided by protein “breathing”—dynamic and rapid fluctuations among the conformational microstates that naturally occur within a population of protein molecules (Somero et al. 2017). TMAO stabilizes the protein and reduces the extent of breathing. The tagging reagent thus has reduced access to buried sulfhydryl groups. Urea, in contrast, opens up the protein and facilitates access to -SH groups. When a 2:1 ratio of urea: TMAO is present, which is the ratio of these two solutes in shallow-living cartilaginous (chondrichthyan) fishes, the rate of labeling does not differ from the control condition (neither urea nor TMAO). These data provide a clear illustration of how stabilizers and denaturants vary in their effects on the stability–flexibility relationship in proteins and show how the influences of different solutes can be counteracting. Thus, at the physiological concentration ratio in shallow-living species of sharks, skates and their relatives, the intrinsic stability of the protein is not affected by the presence of both a denaturant and a stabilizer.

As a side note, the question of how urea and TMAO offset each other’s effects on proteins has been subject to recent study using a wide range of techniques (for review, see Gao et al. 2017b). Direct interactions between TMAO and urea appear to be rare, and the counteraction phenomenon likely is mediated through the different effects of the two osmolytes on water structure, notably strengths of hydrogen bonding relationships. TMAO is a potent water-structurer; its oxygen hydrogen bonds to water more strongly than the bonding found in water–water hydrogen bonding (Yancey and Siebenaller 2015). When surrounded by its highly organized hydration shell, TMAO is able to penetrate the organized water surrounding the protein only very poorly (Auton et al. 2011; Bolen and Rose 2008; Street et al. 2006). Urea hydrogen bonds well to water and to the surface of a protein or nucleic acid. Counteraction is conjectured to arise from the ability of TMAO to increase the strengths of hydrogen bonding between water molecules and between urea and water, leading to a decrease of hydrogen bonds between a macromolecule and urea. In effect, TMAO reduces the ability of (hydrated) urea to enter the hydration shell around the protein.

At this juncture it seems pertinent to mention again that stabilization per se is not necessarily advantageous; this point was briefly raised in discussion of compatibility of solutes. Thus, in the absence of a perturbant like urea, stabilizers like TMAO can have negative effects on biochemical systems, for example, by reducing the rate of enzymatic activity (see Yancey and Siebenaller (2015) for a discussion of these types of effects). The basic point about excessive stabilizing effects from TMAO or other stabilizers is that the optimal flexibility-stability balance is upset, much as would be the case if only a perturbant like urea were present in the system.

**Fig. 2:**
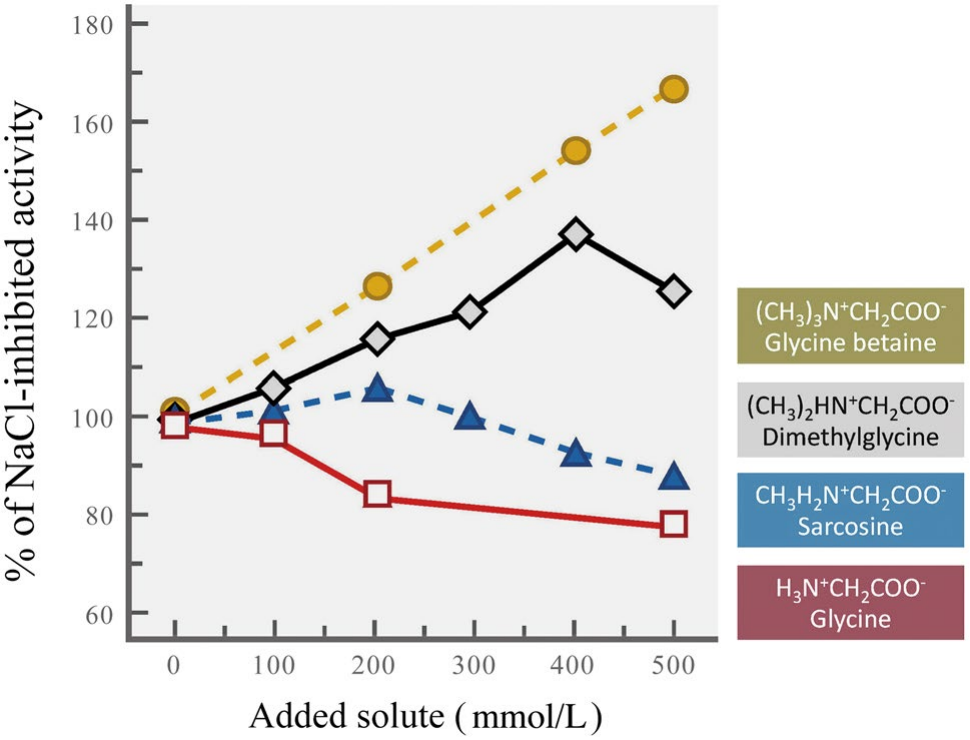
Efficacies of differently methylated forms of glycine in offsetting salt-induced inhibition (300 mol/L NaCl) of an enzyme (malate dehydrogenase from barley). Activation rises as additional methyl groups are added. (Figure redrawn after Pollard and Wynn-Jones 1979)

Another illustration of how organic osmolytes differ in their stabilizing properties—and how a single type of small organic molecule can be tuned to have different stabilizing potential, is given in Fig. 2, which illustrates the abilities of glycine and differently methylated variants of glycine to offset salt-induced inhibition of an enzyme (malate dehydrogenase (MDH) from barley) (Pollard and Wynn-Jones 1979). With increasing amounts of methylation of the amino group, the activating potential of the glycine derivative increased. Similarly, increased methylation was found to enhance the ability of these methylammonium solutes to foster reactivation of a denatured protein (trypsin) (Levy-Sakin et al. 2014). This tuning of stabilizing ability through changes in degree of methylation illustrates one mechanism that could be used to alter the stabilizing potential of the osmolyte pool of the cell. Importantly, the stabilization potential could be modified without changing the total osmolality of the fluid, i.e., without changing the osmotic balance of the organism.

Modulation of the stabilizing potential of the osmolyte pool is relevant not only in short term acclimatization, as might occur, for example, by tide-pool invertebrates during the tidal cycle, but also in longer-term, multi-generational responses such as those made to counteract effects of rising global temperatures. Here, adaptive changes of extrinsic factors like osmolyte systems may be of critical importance because evolution of intrinsic properties of macromolecules, e.g., thermal stability, may not be rapid enough to ensure survival in a warming world.



As mentioned above, especially strong stabilization can be achieved by fabricating relatively large osmolytes from individual components that themselves have stabilizing potential. Here, a good example is glycerylphosphorylcholine (GPC), which is a urea-countering osmolyte that is accumulated in the inner medulla of the mammalian kidney where urea concentrations may rise to high levels (Yancey 2005). GPC is also important in adaptation to extreme depth in certain marine invertebrates, as discussed later (Yancey 2020). GPC is composed of a trio of protein stabilizers: the methylammonium solute choline, the stabilizing phosphate anion, and glycerol, which has widespread stabilizing effects. The effectiveness of GPC as a stabilizer suggests that laboratory synthetic procedures that can fuse together two or more stabilizing osmolytes (or their stabilizing components like trimethylammonium moieties) could yield novel and powerful stabilizers for biotechnological purposes. I return to this point near the end of this review.

Another key point about osmolyte-macromolecule interactions is that a given osmolyte typically has similar effects on proteins, nucleic acids, and lipoprotein membranes, a fact which suggests some common mechanism of stabilizing or destabilizing effects. Here, as alluded to above in the case of TMAO and urea effects, the structure of water is the common element underlying many of these effects. Through having similar effects on proteins, nucleic acids, and membranes, a change in intracellular osmolyte composition is likely to have global effects on the large molecular systems of the cell.

A further key point about osmolyte–macromolecule interactions is that a given osmolyte commonly can offset perturbation caused by different types of stress. A structure-stabilizing organic osmolyte is likely to protect against extremes of temperature, osmolality, and hydrostatic pressure. In addition to their roles in maintaining (or restoring) the appropriate stability/flexibility balance of macromolecules and membranes, certain osmolytes can provide additional benefits to the organism. Some protect against oxidative stress by facilitating degradation of reactive oxygen species (ROS) (Yancey 2005; Yancey and Siebenaller 2015). Other functions of osmolytes include predator repulsion, sulfide detoxification, and provision of energy reserves (Yancey 2005).

I now turn to the examination of effects of two key abiotic stressors, temperature and hydrostatic pressure, on proteins, nucleic acids, and membranes and show how adaptive changes in osmolyte composition and concentration overcome many of the challenges posed by these stressors.

## Temperature

### The pervasive effects of temperature and the protective effects of osmolytes

Almost every cellular structure and process found in living systems is influenced by changes in temperature (Somero 2020; Somero et al. 2017). Several basic categories of temperature effects are commonly studied by physiologists and biochemists, including temperature’s effects on rates of activity, e.g., on velocities of enzyme-catalyzed reactions; effects on the biochemical composition of the organism, e.g., relative amounts of difference types of enzymatic pathways; and influences on the structural stabilities of large molecular systems, all of which depend on thermally labile weak-bonded interactions. Here, attention will focus entirely on this third broad category of temperature effects. I first examine how temperature perturbs higher-order structures of proteins, nucleic acids and membranes and then, analyze how adjustments in the solutions bathing these large molecular systems help to maintain the biologically appropriate balance between stability and conformational flexibility.

Our discussion of adaptive changes in osmolyte systems will be structured around the following questions. First, does the macromolecular stabilizing power of the intracellular osmolyte pool vary with evolutionary adaptation temperature and with the recent thermal exposure of the organisms (acclimatization effects)? Second, in modulating the stabilizing power of the osmolyte pool, do adaptive changes involve alterations in the types of osmolytes used, changes in their absolute or relative concentrations, or a combination of both of these strategies? This second question will receive considerable attention when we examine the osmolytes found in extremophile organisms. Here, novel types of osmolytes have been developed during evolution to confer extraordinary stabilization capacity on the osmolyte system.

### Extremophiles: evolution of “extremolytes” and “thermolytes”

An appropriate way to initiate this analysis then is through examining thermophilic (heat-loving) micro-organisms, some of which thrive at temperatures as high as ~ 122 °C (Clarke 2014; Kashefi and Lovley 2003). To provide context for this analysis, a brief examination of the thermal tolerance ranges of different groups of thermophiles is appropriate. Although different authors have used different criteria and terminology for characterizing the degrees of thermophilia in bacteria and archaea, a commonly used set of distinctions is as follows (Koul et al. 2021): *Moderately thermophilic*: survival up to 45 °C; *extremely thermophilic*: survival up to 80 °C; and *hyperthermophilic*: survival at 80 °C and higher. Some authors group thermophiles into only two categories: *thermophiles* are organisms with optimal growth temperatures (OGTs) between 65 and 80 °C; *hyperthermophiles* have OGTs above 80 °C (Santos and da Costa 2002). The upper thermal limit for Bacteria is ~ 100 °C, approximately 20 °C lower than the limit for Archaea, ~ 122 °C (Clarke 2014). The majority of hyperthermophiles belong to the domain Archaea. In general, hyperthermophilic species of both domains have been isolated mostly from marine geothermal areas, although some species are also found in continental hot springs (Empadinhas and da Costa 2006). As we will see below, these differences in upper thermal tolerance limits are associated with novel adaptive differences in the thermoprotective osmolytes accumulated by these different groups of heat-tolerant microbes.

Studies of hyperthermophiles have shown that the cellular osmolyte pool contains osmolytes that are extraordinarily stabilizing of protein structure and that the concentrations of these osmolytes vary with culture temperature. In some cases, stabilizing osmolytes found in other species, including certain marine animals and algae, are present at high concentrations along with the more stabilizing archaeal osmolytes. The protein-stabilizing amino acids aspartate and glutamate are examples of taxonomically widely occur protein stabilizers (Bowlus and Somero 1979; Empadinhas and da Costa 2006). In other cases, the thermoprotectant molecules found in hyperthermophiles appear to be evolutionary inventions that suggest a need for more stabilizing osmolytes than can be found in the osmolyte pools of mesophiles. Thus, as I point out below, commonly occurring osmolytes like amino acids and glycine betaine, although stabilizing of macromolecules in many cases, may not possess great enough stabilizing power, at least at concentrations consistent with cellular physiology, to protect macromolecules of hyperthermophiles.

**Fig. 3:**
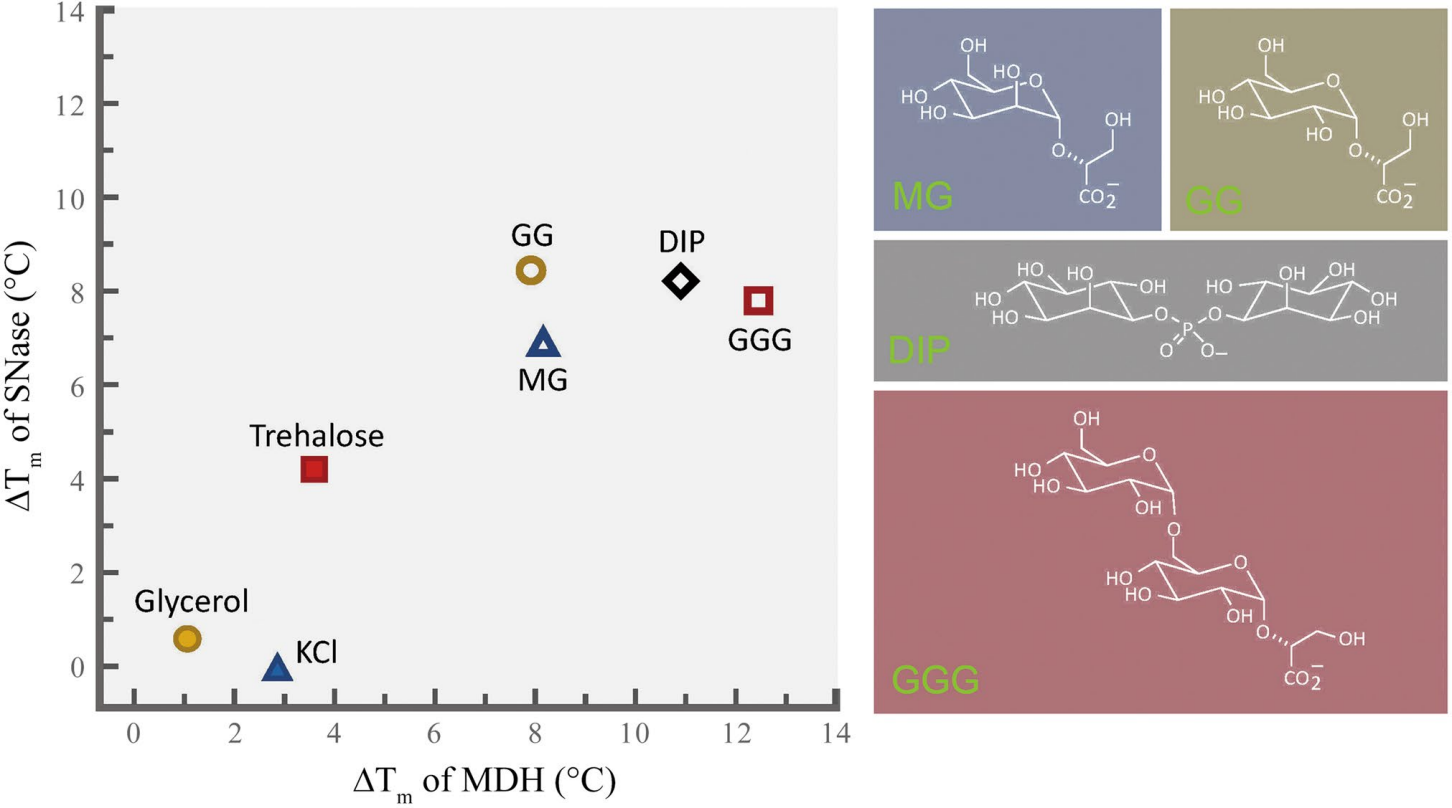
The efficacies of different organic osmolytes in stabilizing the structures of malate dehydrogenase (MDH) and staphylococcal nuclease (SNase). Osmolyte concentrations were 0.5 mol/L except for GGG, which was 0.4 mol/L. Chemical structures of the extremolytes, MG (mannosylglycerate), GG (glucosylglycerate), DIP (di-myo-inositol 1-3’phosphate) and GGG (α(1,6)glucosyl-α(1,2) glucosylglycerate) are shown to right of the graph. (Figure modified after Lamosa et al. 2013)

The extremely powerful thermoprotectants found in hyperthermophiles include the molecules shown on the right of Fig. 3. These powerful thermoprotectants all are polyhydroxylated molecules that bear a net negative charge. This charge is likely to be neutralized by the high concentrations of potassium ion commonly found in cells of extremophiles (Empadinhas and da Costa 2006). The stabilizing power of these anionic osmolytes, which are sometimes referred to as “*thermolytes*” (Esteves et al 2014) or as members of the “*extremolytes*” (Becker and Wittman 2020), relative to less potent thermoprotective osmolytes is shown in Fig. 3. All of the anionic osmolytes increased the melting temperatures of the two enzymes examined, staphylococcal nuclease (SNase) and malate dehydrogenase (MDH) (Lamosa et al. 2013). The similar stabilizing effects of the different osmolytes on these two proteins speak to the common effects of an osmolyte across different protein systems.

Concentrations of these powerful thermoprotectants have been shown to vary with culture temperature, with rising temperatures promoting large increases in the concentrations of thermolytes (Esteves et al. 2019). For example, the hyperthermophilic marine archaeon *Pyrococcus furiosus* increased concentrations of DIP and MG by 5- and sevenfold, respectively when culture temperature was raised from 90 to 97 °C. In parallel with these increases in concentrations of extreme thermoprotectants, some of the most prevalent organic osmolytes found under lower temperature culture (90 °C) decreased in concentration; for example, aspartate concentrations fell by ~ 50% as culture temperature increased (Esteves et al. 2019). This study is a clear indication of how the cellular osmolyte pool is closely modulated to “titrate” protein stability in the face of thermal stress. It is noteworthy that glycine betaine, which is a major osmolyte in many mesophilic species and some thermophiles, is not detected in (hyper)thermophilic bacteria or archaea, where the more strongly thermoprotective anionic thermolytes like DIP play dominant roles (Empadinhas et al. 2007; Lamosa et al. 2013; Santos and da Costa 2002). DIP’s role as a potent thermoprotectant is suggested by its occurrence only in microbes with optimal growth temperatures above 60 °C (Borges et al. 2010). Whereas DIP appears to be the thermoprotectant of choice in most hyperthermophilic archaea, mutants of the archaeon *Thermococcus kodakarensis* (optimal growth temperature of 85 °C) in which DIP synthesis was blocked nonetheless survived supraoptimal temperatures (93.7 °C) by accumulating high concentrations of aspartate (Borges et al. 2010). This study also showed that aspartate levels rise under osmotic stress, an illustration of the multi-tasking capabilities of many organic osmolytes. Similarly, studies of the hyperthermophilic archaeon *Pyrococcus furiosus* (OGT between 90 and 95 °C, depending on strain) demonstrate the dual roles of strong thermoprotectants like DIP and MG in responding to stresses from extremes of temperature and salinity (Esteves et al. 2014, 2019). MG and DIP are both strong thermoprotectants of proteins (Fig. 3). However, DIP is somewhat more effective than MG and is preferentially synthesized under heat stress. In contrast, the synthesis of MG is more responsive to salinity stress. However, DIP and MG can substitute for each other when conditions demand this. Mutant strains of *P. furiosus* that lack capacities for producing either DIP or MG accumulated the thermolyte they could produce when challenged by supraoptimal temperatures (Esteves et al. 2014). This finding is consistent with the discovery that a few extremely thermotolerant archaea and bacteria lack the biosynthetic pathways needed for DIP synthesis and accumulate MG under heat stress (Esteves et al. 2014).

In the context of the varied osmolyte compositions observed in different bacteria and archaea, it bears noting that the osmolyte pools found in microbes cultured in the laboratory reflect both the biosynthetic capacities of the microbe and the composition of the growth medium (Esteves et al. 2014; Santos and da Costa 2002). Osmolytes can be synthesized de novo or taken up from the medium, so a given microbe may exhibit very different osmolyte pools when culture conditions are varied. And, as shown by the study of *T. kodakarensis* (Borges et al. 2010), genetic variation among microbes of a single taxonomic group can lead to variation in the pattern of osmolyte composition. Generalizations about the typical osmolyte compositions of microbes thus must be avoided or at least made with care, including consideration of culture conditions and genetic strain of the organism.

Mesophilic and moderately thermophilic marine bacteria appear to lack these very strong thermoprotectants and rely instead on a variety of amino acids, amino acid derivatives, and carbohydrate osmolytes (notably trehalose) (Santos and da Costa 2002). However, extremely thermophilic bacteria have been shown to accumulate certain of these strong thermoprotectants under culture at high temperatures. For example, a thermotolerant (60 °C OGT), radiation tolerant, and halotolerant bacterium (*Rubrobacter xylanophilus*) isolated from a polluted fresh water source produced DIP and MG (Empadinhas et al. 2007).

To summarize the likely roles of thermoprotectant extremolytes in archaea and bacteria, we can conjecture that the stabilizing potential of the osmolyte pool for protection of cellular proteins and, by implication, nucleic acids (see below) is closely regulated to ensure that stabilities are defended within ranges that are optimal for molecular function. Despite the fact that proteins of extremophilic microbes typically exhibit high intrinsic thermal stabilities, to ensure that optimal states of conformational flexibility are retained across the full range of operating temperatures of a species, precise regulation of the osmolyte pool is essential.

### Mesophiles: adaptive roles of “normal” osmolytes at high- and low temperatures

In view of the adaptive patterns found in (hyper)thermophilic archaea and bacteria, we can ask if osmoconforming marine animals and algae exhibit similar temperature-related patterns of osmolyte accumulation. Although studies of temperature-dependent accumulation of stabilizing osmolytes in marine eukaryotes are in their infancy, existing data on marine invertebrates are consistent with the conjecture that the types of osmolytes that are accumulated, as well as their cellular concentrations, reflect the level of temperature stress the organism faces. Unlike hyperthermophilic archaea, however, marine invertebrates appear to accumulate solutes that are common components of central pathways of cellular biochemistry, and which serve a wide variety of functions in the cell; unique micromolecules selected entirely (or largely) for their thermoprotective abilities seem not to have evolved in these mesophilic species.

Two studies of osmolyte pools in intertidal molluscs shed insight into the roles of adaptive changes in osmolyte composition in the face of temperature change. In gill tissue of the cool-temperate ribbed mussel *Mytilus californianus*, the greatest up-regulation of an organic osmolyte under elevated temperatures in the field was taurine, a relatively weak protein stabilizer (Yancey et al. 1982) whose concentration rose about two-fold (increase of ~ 45 mmol/kg) with rising field temperature (Gleason et al. 2017). Glycine betaine (GB), a stronger protein stabilizer than taurine, increased by about 20 mmol/kg, but this increase did not reach statistical significance. To maintain osmotic balance in the face of upregulation of these two stabilizing osmolytes, concentrations of less stabilizing amino acids, e.g., glycine, were reduced. A recent study of the extremely thermophilic snail *Echinolittorina malaccana*, which withstands temperatures slightly over 55 °C, revealed that foot tissue accumulated high levels of GB as exposure temperatures increased from 25 to 52 °C (Chen et al. 2021). Under heat stress, the concentrations of GB in foot tissue of this thermophilic snail reached much higher values (~ 90 mmol/kg tissue) than those characteristic of less thermally tolerant species. The concentrations of two other methylammonium solutes, choline and carnitine, also rose significantly with rising temperature, further suggesting the importance of methylammonium osmolytes in enhancing macromolecular stability. In addition, even at non-stressful temperatures for this species (25 °C), GB and many amino acids were present at higher levels than typically found in marine invertebrates. The majority of amino acids did not change concentration with temperature, although a significant increase was seen for glutamate, a protein-stabilizing osmolyte (Borges et al. 2010; Bowlus and Somero 1979), and serine, which can stabilize membranes (Vance and Tesseva 2013). Chen et al. (2021) suggested that high constitutive concentrations of amino acids and GB in this species may reflect a pre-adaptation for tolerating episodic extreme heat stress.

Whereas results of these two studies of marine molluscs are consistent with roles of organic osmolytes as thermoprotectants in marine invertebrates, several caveats must be kept in mind. One is that the organic osmolyte pools may vary among tissues within an organism (Yancey et al. 1982). Thus, caution must be taken in making interspecific comparisons that involve different tissues in different species, e.g., gill tissue in work by Gleason et al. (2017) and foot tissue in the study of *E. malaccana* (Chen et al. 2021). A second note of caution concerns the precise role or roles of the observed changes in osmolyte concentrations (Yancey 2005). As mentioned at several junctures in this review, a given osmolyte may play several physiologically important roles. Taurine is a good example, for it may contribute (perhaps indirectly) to reduction of stress from reactive oxygen species as well as serving as an osmolyte (Yancey 2005). Rising taurine levels with increased cell temperature could reflect a stronger anti-oxidant defense as well as (or instead of) an effort to protect macromolecular stability. Lastly, changes in the micromolecular composition of a tissue can result from transitions between normoxic and hypoxic conditions (Haider et al. 2020). Investigating the role of temperature-dependent accumulation of organic osmolytes thus requires that multiple variables be incorporated into the experimental design and that interpretation of results take into account diverse physiological roles of the osmolytes that change concentration.

In conclusion, further exploration of high temperature acclimatization on osmolyte patterns in marine organisms is clearly warranted to further clarify the nature and importance of these responses to rising temperature. Notably, such information could provide important insights for predicting how rising temperatures occurring with global climate change might be accommodated through adjustments in the osmolyte pools of these organisms. Acclimatization-induced adjustments in the stabilizing potential of the cellular fluids might facilitate tolerance of rising temperatures even if these temperature increases occur at a rate that far out paces the ability of slower evolutionary processes to adapt the intrinsic stabilities of macromolecules.

The roles of adaptive changes in osmolyte pools under cold stress have received study in a few marine species, albeit the bulk of this type of research has been done with terrestrial organisms like cold-tolerant insects (Duman 2015; Toxopeus and Sinclair 2018). Because higher orders of macromolecular structure and the biophysical properties of membranes can be disrupted by cold as well as by heat, it is relevant to consider the roles that osmolytes might play in cold tolerance, in addition to preventing the formation of ice in biological fluids.

A study by Kennedy et al. (2020) on the intertidal mollusc *Mytilus trossulus* sought to identify adaptive (cryoprotective) changes in osmolyte systems related to tolerance of low, potentially freezing temperatures. They observed significant effects of season and acclimation salinity on lower lethal temperature (LLT_50_) and the concentrations of major organic osmolytes. High salinities reduced LLT_50_ and elevated concentrations of taurine, glycine, TMAO, and alanine, consistent with a cryoprotective effect of these osmolytes. Mussels from winter populations also had low LLT_50_ values and high concentrations of these same four osmolytes. The authors conjecture that the elevated concentrations of organic osmolytes might have two beneficial (cryoprotective) influences on cold tolerance. First, due to colligative effects, accumulating higher concentrations of osmolytes and thereby elevating osmolality of cellular water would reduce the danger of intracellular ice formation. Second, the favorable effects of these osmolytes on protein stability and membrane biophysical state could facilitate physiological function at the extreme low end of the thermal tolerance range. The authors also point out that studies of this type could help in the identification of osmolytes that might have biomedical relevance in the context of low-temperature storage of tissues and cells. 

Osmolyte stabilization of macromolecular systems may also be important in osmoregulating species like marine bony fishes, which are hypo-osmotic relative to seawater. Thus, the occurrence of high concentrations of TMAO in many Antarctic and Arctic bony fishes may reflect a mechanism of cold tolerance (Raymond 1998; Raymond and DeVries 1998). In Antarctic notothenioid fishes, muscle TMAO levels in some specimens collected in McMurdo Sound, where water temperatures remain near freezing throughout the year, were as high as ~ 150 mmol/kg (Raymond and DeVries 1998). In contrast, the cold temperate notothenioid *Notothenia angustata* from New Zealand waters had muscle TMAO levels of ~ 50 mmol/kg. Muscle of several species of Arctic fish had TMAO levels up to ~ 70 mmol/kg and in some cases (rainbow smelt) concentrations varied significantly with season, rising from < 10 mmol/kg in summer to 50–60 mmol/kg in winter (Raymond 1994). Furthermore, compared to fishes from lower latitudes, some of these polar fishes also had relatively high tissue and blood urea concentrations. However, these concentrations were much lower than TMAO concentrations, a finding that contrasts with the ~ 2:1 urea:TMAO ratio of shallow-living cartilaginous fishes. The functions of high TMAO concentrations in polar fishes remain a matter of debate (Raymond and DeVries 1998). Through their colligative effects, they would reduce dangers of ice formation, but their contribution to freezing resistance would be much less than that due to macromolecular thermal hysteresis (“antifreeze”) proteins. TMAO might also offset inhibition of cellular biochemical processes by the relatively high concentrations of inorganic ions (chiefly K^+^ and Na^+^) found in these species, an effect of methylammonium solutes shown in Fig. 1. TMAO might also function to reduce low temperature damage to proteins. For example, low temperatures can disrupt hydrophobic interactions and foster unfolding of the native conformation of a protein or nucleic acid. Thus, the structure-stabilizing effects of TMAO could be beneficial to macromolecular stability at low as well as at high temperatures. The role of TMAO and other methylammonium compounds as cryopreservatives merits additional study. 

### Universality of thermoprotective osmolyte effects: proteins, nucleic acids, and membranes

#### Nucleic acids

The above discussion of amelioration of thermal perturbation of macromolecules by osmolytes has centered either explicitly or implicitly on proteins. This focus reflects the fact that most studies of temperature effects on macromolecule-osmolyte interactions have focused on proteins; nucleic acids have yet to receive widespread study in this context (Leamy et al. 2016). However, the patterns of intrinsic and extrinsic adaptive changes demonstrated for proteins apply as well to nucleic acids, at least for certain classes of RNAs for which alterations in secondary structure are known to have wide functional importance (Mortimer et al. 2014). For example, secondary structures of mRNAs (hairpin loop structures, etc.), like the conformations of proteins, must be able to undergo reversible changes in shape to enable effective regulation of mRNA-based processes like initiation of translation, modulation of translation rate, and splicing activity. Having the right poise between stability and flexibility again is critical. Thus, as in the case of orthologous proteins from species adapted to different temperatures, the intrinsic stabilities of secondary structures of mRNAs, as measured by the free energy change that occurs during formation of these structures, exhibit a clear signal of temperature adaptation. For instance, the intrinsic stabilities of secondary structures of mRNAs encoding cytosolic malate dehydrogenases (cMDH) of marine molluscs adapted to temperatures from approximately − 2 °C (Southern Ocean) to ~ 55 °C (Eastern Pacific rocky intertidal zones) vary regularly with evolutionary adaptation temperature (Liao et al. 2021), reflecting the pattern of adaptation found in the cMDH protein as well (Dong et al. 2018; Liao et al. 2019).

Adaptive modulation of the organic osmolyte pool could play major roles in ensuring that the needed flexibility–stability balance of mRNA secondary structure that has developed during long-term evolutionary processes is maintained in the face of rapid change in cell temperatures. The reduced stability of RNA secondary structure at rising temperatures could be offset by accumulation of osmolytes that stabilize RNA higher order structures. TMAO once again looms as an important candidate for this stabilization function, although its stabilizing effects on secondary structure may be smaller than those found on RNA tertiary structure (Denning et al. 2013; Leamy et al. 2016). Thus, TMAO effects on secondary structure have been reported to range from slightly destabilizing to strongly stabilizing, whereas TMAO is consistently stabilizing of RNA tertiary structure (Gao et al. 2017b).

**Fig. 4:**
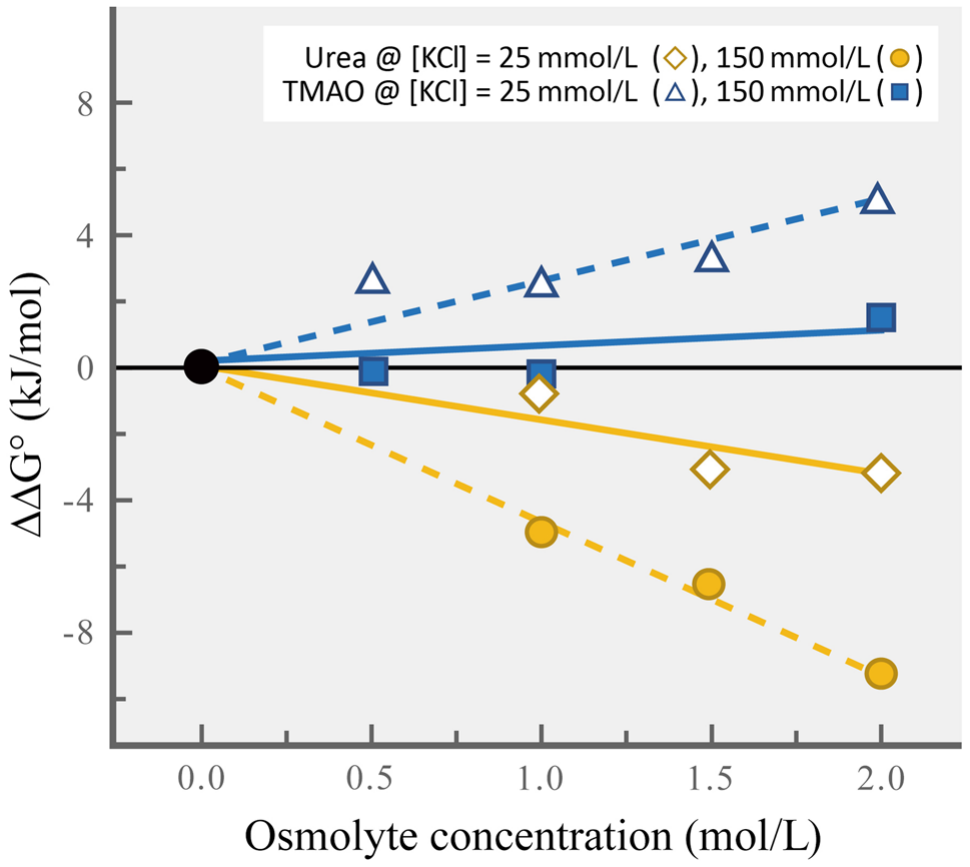
The effects of TMAO, urea, and KCl concentration on the stability of an RNA thermometer. The filled black circle indicates no added solutes to the buffer mixture. TMAO and urea were separately added to solutions that contained either 25 mmol/L or 150 mmol/L KCl. (Figure redrawn after Gao et al. 2017a)

An interesting example of osmolyte effects on stability of RNA secondary structure is given by a study of effects of TMAO and urea on a bacterial RNA thermometer, the *Salmonella* 4U RNA thermometer (Fig. 4; Gao et al. 2017a). This RNA thermometer consists of a 34 nucleotide hairpin loop located near the 5′ end of the RNA sequence. The structural transition undergone by the thermometer at high temperatures activates a heat-shock gene that is part of the bacterium’s CSR. TMAO increased the stability of secondary structure, as shown by an increase in the positive change in free energy (ΔΔ*G*°) that accompanies unfolding of the hairpin loop of the thermometer. This increase in thermodynamic stability was proportional to the concentration of TMAO and was stronger in the presence of low (25 mmol/L) than of high (150 mmol/L) concentrations of KCl. In contrast to the effects of TMAO, urea destabilized the hairpin loop in a concentration-dependent manner; the unfolding of structure occurred with an increasingly negative value of Δ*G*° as urea concentration rose. The destabilizing effect was greater at high than at low concentrations of KCl, much as TMAO’s stabilizing effect was lower at the higher KCl concentration. The destabilizing effects of KCl may derive from the chloride ion, not the potassium ion (see Bowlus and Somero 1979). Whatever their mechanistic bases, the effects of an inorganic salt on the osmolyte-dependence of the folding-unfolding equilibrium sound a cautionary note about the design of experiments and interpretation of results in solute-macromolecular studies. However, the key message of the study of Gao and colleagues is that the adaptive importance of accumulation of methylammonium solutes (TMAO and GB) in species like marine elasmobranchs that employ high concentrations of urea as an osmolyte can be seen to benefit nucleic acids as well as proteins. A clear example of urea and TMAO counteraction on RNA is given by studies of stability of tertiary structure of a transfer RNA (tRNA) of the bacterium *E. coli* (Gluick and Yadav 2003). Urea caused a loss of tertiary folding whereas TMAO favored the folded state. Together, at a 2:1 concentration ratio of urea:TMAO, the tRNA’s stability was similar to that found in buffer lacking TMAO and urea.

Mechanistically, the different effects of TMAO and urea reflect phenomena previously observed with proteins. In the latter molecules, TMAO is strongly excluded from the protein surface due to effects mediated by water structure around TMAO and near the backbone peptide bonds between residues of a protein (Auton et al. 2011). In RNA too, TMAO is strongly excluded from the nucleic acid surface, particularly in the case of the phosphate group, and it favors burial of the RNA backbone in tertiary structures (Gao et al. 2017a). However, there is some evidence from in silico analyses that the phosphate group of RNA can alter the pK_a_ of TMAO and thereby change its ability to interact with RNA (Denning et al. 2013); the biological implications of this finding remain to be determined. Urea by contrast interacts with all components of RNA (nucleobase, ribose, and phosphate), favoring an opening-up of RNA structure, much as occurs in proteins.

The similarities in the building blocks of RNA and DNA suggest that common osmolyte effects are likely to be found in both classes of nucleic acids. In fact, available data support this expectation. In a study of a DNA hairpin loop, Patra et al. (2019) found that TMAO favored the closed state of the loop, whereas urea destabilized the closed conformation. Rising temperature would be expected to favor the open conformation of the loop, but the stabilizing effect of TMAO would be expected to offset this perturbation. Further examination of osmolyte effects on nucleic acids are warranted, to see how osmolytes with known differences in abilities to stabilize proteins (including the potent extremolytes shown in Fig. 3) affect stabilities of higher orders of nucleic acid structures.

#### Membranes

The effects of different organic osmolytes on cellular membranes have received relatively little examination to date and no studies to my knowledge have evaluated osmolyte effects on true biological membranes, which of course are complex, highly organized mixtures of proteins, phospholipids, cholesterol, and carbohydrates. Some experimentation has utilized synthetic membranes that lack proteins. Other approaches to the question have employed an in silico method, molecular dynamics simulations (MDS), but this work has focused on relatively simple lipid-based systems rather than biological membranes (Maiti and Daschakraborty 2021). Furthermore, these in vitro and in silico studies have employed concentrations of the osmolyte of interest, TMAO, that greatly exceeded physiological concentrations. Thus, whereas results may point to important directional trends, interpreting the data quantitatively in the context of biological systems is impossible at the moment. Nonetheless, despite a relative lack of data, observations made with these simplified artificial study systems suggest that membranes exhibit responses to stabilizing and, by inference, destabilizing osmolytes that are directionally similar to effects seen for proteins and nucleic acids. Stabilizers of proteins and nucleic acids also have stabilizing effects on biophysical properties of membranes. Thus, adjustments in concentrations of membrane-stabilizing osmolytes could offset the influences of temperature on membrane fluidity and ability to form non-bilayer structures.

In a study utilizing synthetic lipid bilayer membranes and multilamellar vesicles, Manisegaran et al. (2019) found that TMAO had strong effects on the gel-fluid transition temperature and on the order of lipid acyl chains within the synthetic membrane. TMAO stabilized the gel state, as seen by increases in the gel-to-fluid transition temperature with rising TMAO concentrations (3 or 6 mol/L). The order of lipids within the membrane also increased in a concentration-dependent manner with addition of TMAO. The in silico MDS study of Maiti and Daschakraborty (2021) found similar TMAO-membrane interactions. If we extrapolate these findings to biological membranes, these data suggest that functionally disadvantageous elevations in fluidity of membranes caused by increases in temperature could be offset by accumulating higher concentrations of a stabilizing osmolyte like TMAO. Conversely, decreasing cell temperatures might lead to acclimatizations that reduce the stabilizing potential of the osmolyte pool, possibly through replacing a highly stabilizing osmolyte like TMAO or GB with a less stabilizing amino acid osmolyte.

These studies also showed that the mechanisms by which TMAO and, by extension, other stabilizing osmolytes like GB exert their stabilizing effects are basically the same water-structure-dependent mechanisms that account for stabilization of protein and nucleic acid structures. Most significantly, TMAO was found to be strongly excluded from the membrane surface (Maiti and Daschakraborty 2021), much as has been observed in many studies of TMAO-protein interactions (Auton et al. 2011). As stated earlier, the preferential exclusion of stabilizing osmolytes from macromolecular (or membrane) surfaces is a key factor in the thermodynamic explanation of osmolyte effects (Auton et al. 2011). The strong water-structuring ability of TMAO reduces its ability to penetrate the hydration layers that surround large biomolecules, including membranes. In the case of lipid bilayers, TMAO reduces the hydration level of head groups, which leads to a compaction of membrane structure and increased order of acyl chains (Maiti and Daschakraborty 2021). TMAO also leads to stronger interactions between adjacent bilayers, bringing them into closer proximity (Sukenik et al. 2017). This effect is also a consequence of TMAO’s exclusion from the surfaces of lipid headgroups.

Studies of effects of other types of organic osmolytes on synthetic membranes (monolayers and bilayers) have revealed effects that differ in important ways from those found with TMAO. For example, studies of the osmolytes ectoine, hydroxyectoine, and ß-hydroxybutyrate showed that these osmolytes (again, used at unphysiologically high concentrations) increased the stability of the gel phase, but in some cases and also increased the fluidity of the liquid–crystalline phase (Herzog et al. 2019). Future studies of membrane-osmolyte relationships should strive to be more biologically realistic through use of membranes isolated from cells rather than prepared de novo from a few types of lipids, and by conducting the studies under conditions of osmolyte concentration that reflect the in vivo state.

Lastly, it should be emphasized that the properties of the liquid–crystalline state and the propensity for forming nonlamellar structures are strongly determined by the lipid composition of natural membranes, including acyl chain and head-group compositions (Hazel 1995; Logue et al. 2000). These compositional changes can occur over time courses of relevance for marine species that face relatively rapid changes in body temperature, such as commonly occur during tidal cycles (Williams and Somero 1996). These time courses are similar to those that may characterize adaptive changes in osmolyte systems. Thus, osmolyte modulation of the biophysical properties of membranes may occur concurrently with adaptive changes in lipid composition and contribute to a multi-faceted set of stress responses that function to maintain the optimal biophysical state of cellular membranes.

### Hydrostatic pressure

#### Hydrostatic pressure effects on biochemistry: volume changes and their sources

The conclusions reached in the analysis of micromolecule–macromolecule interactions under different thermal conditions are, by and large, applicable to hydrostatic pressure effects as well. For both stressors, the perturbation induced by the stressor results principally from its effects on higher order macromolecular structures stabilized by non-covalent bonds. In the case of hydrostatic pressure, perturbation occurs when an alteration in system volume takes place during the biochemical process. These effects reflect Le Châtelier’s Principle: pressure enhances processes that involve decreases in volume and inhibits those that are accompanied by a volume increase (Somero 1990; Winter 2019). Tolerance of high pressure depends on both intrinsic adaptation of macromolecules and the stabilizing effects of organic osmolytes. A clear signature of adaptation to pressure is found in protein systems: orthologs of deep-living species are more resistant to perturbation by pressure relative to orthologs of shallow-occurring species (Dahlhoff and Somero 1991; Yancey 2020; Yancey and Siebenaller 2015). However, perturbation is not completely eliminated by these intrinsic adaptations. Thus, with both stressors, perturbation requires some amelioration by changes in the osmolyte composition of the cellular solution.

Here, as in the case of temperature, the system must be appreciated as one that includes water as well as the osmolytes, macromolecules, or membranes that are present. Volume changes may derive from alterations in the volume of the large molecular system itself or through changes in hydration that lead to alteration of the volume of water. Thus, for example, the denaturing effect of elevated pressures on proteins reflects in part water re-organization around the amino acid residues that gain exposure to solvent during unfolding. The volume of the water surrounding the exposed residues is less than the volume of the corresponding amount of bulk water in the solution itself. Volume changes can also occur when the structure of a large molecular system itself can be compressed by pressure. The compressive effect of pressure on membranes, in which acyl chains are compacted into a smaller volume serves as a good example of this type of pressure perturbation (Cossins and Macdonald 1989).

#### Depth-related patterns of osmolyte accumulation: the interesting case of TMAO

In view of the interactions among osmolytes, water, and large molecular systems examined in the context of temperature, it would seem likely that certain osmolytes would have significant effects on the pressure sensitivities of these systems as well. Indeed, a fascinating story has developed on this topic, one that provides an exceptionally clear example of the importance of closely regulating osmolyte composition and concentration to confer on macromolecules a fitness for function in a stressful environment. The first major insight into the role of stabilizing osmolytes in deep-living species came from studies by Paul Yancey and colleagues who measured osmolyte concentrations of fishes and invertebrates collected at different depths (Kelly and Yancey 1999; Yancey 2020; Yancey et al. 2002, 2004). TMAO concentrations increased linearly with capture depth in teleost fishes and many invertebrates and represented the largest change in osmolyte concentrations with depth. This finding suggests that TMAO might offset perturbation by pressure, much as it can stabilize proteins against other stressors, notably urea in the case of cartilaginous fishes (Fig. 1; Yancey et al. 1982). A solute that can counteract perturbation by pressure has been termed a *piezolyte,* a term that derives from the Greek word for “squeeze” or “press” (Martin et al. 2002).

**Fig. 5:**
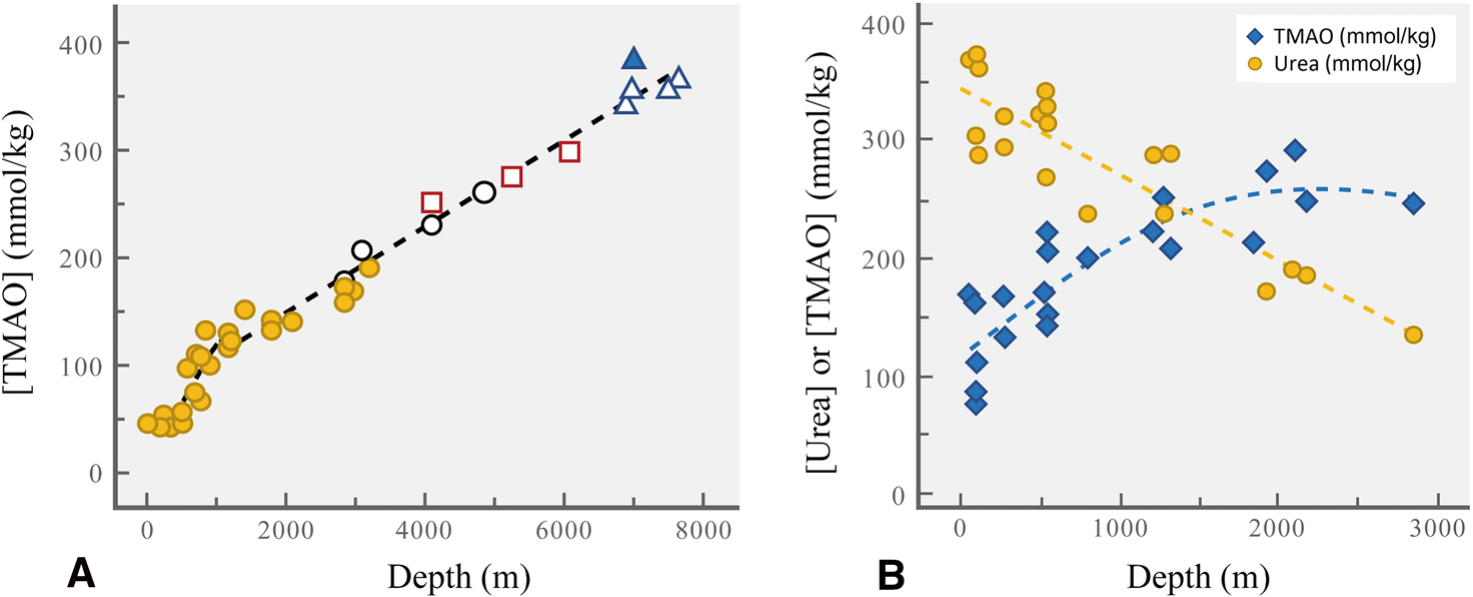
**A** Depth-related concentrations of TMAO in skeletal muscle of marine bony fishes belonging to 9 families. Each point represents a single specimen captured at the depth shown on the *X*-axis. Data for three species captured at multiple depths are indicated by open symbols: the rattail fish *Coryphaenoides armatus* (open circles); the rattail fish *Coryphaenoides yaquinae* (open squares), and the Marianas Trench snailfish, *Pseudoliparis swirei* (open triangles). (Figure redrawn after Yancey 2020). **B** Depth-related patterns of urea and TMAO concentrations in skeletal muscle of 15 species of marine chondrichthyan fishes (sharks, skates, chimeras, and rays). Each point represents either the urea or TMAO concentration from a single specimen. (Figure redrawn after Laxon et al. 2011)

The striking depth-related trend in TMAO concentrations in teleosts is shown in Fig. 5A. This pattern of depth-related accumulation of TMAO provides a clear example of convergent evolution among diverse taxa; fish from different families exhibited essentially the same depth-related trend of rising [TMAO] with increasing collection depth. Note, too, that for a single species captured at different depths (the rattail fishes *Coryphaenoides armatus* (open circles) and *Coryphaenoides yaquinae* (open squares) and the Marianas Trench snailfish, *Pseudoliparis swirei* (open triangles) the concentration of TMAO again reflects the depth of capture. The latter discovery points to a close regulation of TMAO levels as organisms change depth. This regulation may involve a combination of retention of TMAO obtained from the diet (e.g., by preying on other deep-living species rich in TMAO), or de novo synthesis of TMAO. The relative roles of these two sources of TMAO remain to be evaluated and no doubt vary widely among species. Evidence for a critical role of de novo biosynthesis of TMAO in the deep-living snailfish (*Pseudoliparis swirei*) from the Mariana Trench has recently come from an evolutionary genomics study that yielded data consistent with enhanced capacity for TMAO synthesis (Wang et al. 2019).

The accumulation of such high concentrations of TMAO in marine teleost fishes is especially interesting because marine teleosts are characteristically strongly hypo-osmotic to sea water (Yancey 2005). However, the osmolality of the deepest living fishes rises to equal that of sea water because of the high concentrations of TMAO that occur in these species. Thus, for example, in the Kermadec Trench snailfish *Notoliparis kermadecensis* collected near 7000 m depth (filled triangle), TMAO reaches ~ 400 mmol/L and the osmolality of the body fluids reaches 991 mOsm/kg. Here, then, the fish is essentially iso-osmotic with seawater. Yancey and colleagues (Yancey et al. 2014), who discovered this phenomenon, conjecture that osmotic relationships may limit the depth distribution of teleosts. Thus, from available data on observation and capture of marine fishes, no teleost is known to occur below ~ 8000 m, the depth at which a bony fish becomes approximately iso-osmotic with seawater because of rising levels of TMAO. Yancey and colleagues conjectured that fundamental features of the osmoregulatory systems of bony fishes may preclude them from becoming hyper-osmotic to seawater.

Marine cartilaginous fishes also present an interesting story in terms of depth-related osmolyte patterns (Laxson et al. 2011; Yancey and Siebenaller 2015). As shown in Fig. 5B, the canonical 2:1 ratio of urea to TMAO found in shallow living chondrichthyan fishes is not found in deeper-living species. Rather, as [TMAO] rises with increasing depth, [urea] falls, until at a depth close to 1500 m the two counteracting osmolytes occur at approximately the same concentration. At greater depths, [TMAO] exceeds [urea]. Despite the alterations in concentrations of these two osmolytes with depth, the summed concentration of TMAO and urea remains relatively stable across depth (Laxson et al. 2011). Thus, unlike bony fish, chondrichthyans maintain similar osmolalities throughout the water column. Also, unlike bony fish, which occur to depths of ~ 8000 m, cartilaginous fish have not been observed at depths greater than ~ 4200 m (Yancey et al. 2014). The basis of the more restricted depth distribution range of chondrichthyans is not known, but Laxon et al. (2011) conjecture that it could result from limitations in obtaining or releasing osmolytes. In the case of the urea, whose perturbation would add to that from hydrostatic pressure, reducing concentrations below approximately 150 mmol/L might not be possible because of effective urea retention mechanisms in this group of fishes. For TMAO, it is possible that limitations to dietary acquisition or de novo synthesis would place an upper limit on concentration. Whatever the bases of the TMAO and urea concentration limits in these fishes may be, the depth-dependent pattern in the ratio of [TMAO] to [urea] may reflect the need to reduce concentrations of the macromolecule-perturbant urea as perturbation by hydrostatic pressure rises with depth. Reducing [urea] and increasing [TMAO] thus may facilitate retention of the optimal range of macromolecular flexibility across the depth ranges of the species, as discussed below.

### Other known or potential piezolytes: lessons from deep-living amphipods

Unlike bony and cartilaginous fishes, some invertebrates have depth ranges that extend into the deepest trench regions of the ocean, the Challenger Deep of the Mariana Trench (10,991 m) and deepest portions of the Kermadec Trench (10,005 m). Downing et al. (2018) reported on novel depth-related osmolyte patterns in several species of amphipods native to different regions of the water column, ranging from shallow tidal pools to the two deepest trenches. TMAO again showed the largest increase in depth, a trend confirmed by recent findings with hadal amphipods from the Marianas and New Britain Trenches (Liu et al. 2022). However, the broad suite of osmolytes analyzed by Downing et al. (2018) presents a more complete picture of the complex depth-related patterns in a number of osmolytes, most of which have not been examined for piezolyte function. Four other osmolytes also exhibited increases in concentration with depth of collection: two fully N-methylated compounds, proline betaine and glycerophosphorylcholine (GPC), and two non-methylated solutes, glycerophosphorylethanolamine (GPE), and *scyllo*-inositol. As concentrations of these five osmolytes increased with depth, there were corresponding decreases in osmolytes typical of shallow-occurring species: glycine, taurine and glycine betaine. Total osmolality thus is conserved whereas the qualitative composition of the organic osmolyte pool changes markedly. Thus, it appears that solutes having at best weak protein-stabilizing abilities are replaced by osmolytes which, in at least in the cases of TMAO and GPC, stabilize proteins very strongly. Further work is needed to determine how proline betaine, GPE and *scyllo*-inositol influence the pressure sensitivities of macromolecules and membranes. One intriguing finding that may relate to this question is the discovery that the increases with depth in concentrations of GPE, proline betaine, and *scyllo*-inositol occur at higher rates than the rise in TMAO, even though TMAO remains the dominant osmolyte showing increases. These contrasting patterns of accumulation rates with increasing depth might reflect even stronger stabilizing effects by these three osmolytes than afforded by TMAO. Alternatively, as discussed below in the context of pressure-membrane interactions, one or more of these rapidly accumulating osmolytes may be critical for conserving membrane structure at high pressure. For example, *scyllo*-inositol has known effects on membranes and on protein aggregation processes and may therefore be serving different or additional functions from those carried out by TMAO (Downing et al. 2018).

Further exploration of osmolyte systems of bacteria and archaea from different depths also may provide new insights into the types of molecules that serve piezolyte functions. For example, Martin et al. (2002) discovered that β-hydroxybutyrate (β-HB) increased in concentration when cultures of the study bacterium (*Photobacterium profundum*) were taken from pressures of 0.1–28 MPa. At this higher pressure, β-HB became the major osmolyte, reflecting the same type of pressure-related pattern noted for TMAO in fishes. To my knowledge, the potential of β-HB to serve as a stabilizer against pressure remains to be elucidated.

### Protective roles of piezolytes on large molecular systems

#### Proteins

As in the case of temperature, adaptive changes to stress from hydrostatic pressure involve both intrinsic and extrinsic mechanisms (Gibbs and Somero 1990; Yancey 2020; Yancey and Siebenaller 2015). Proteins of pressure-adapted organisms found in the deep sea typically exhibit a reduced sensitivity to elevated pressure compared to orthologous proteins from shallow-living species. However, even the relatively pressure-resistant orthologs of certain enzymes are at least somewhat perturbed by high pressures, so extrinsic factors, namely the accumulation of high concentrations of stabilizing osmolytes (piezolytes) are likely needed to enable effective biochemical functioning at great depths. TMAO is a powerful piezolyte that has been shown repeatedly to offset the perturbing effects of high pressure on enzyme structural stability and function (Yancey and Siebenaller 2015; Yancey 2020). Thus, the depth-related patterns of TMAO concentration illustrated in Fig. 5 reflect the role of this macromolecular stabilizer in facilitating life in the deep sea. Moreover, the abilities of marine teleosts and cartilaginous fishes to vary [TMAO] in a depth-dependent manner suggests that vertical migration in the water column requires adjustment in the osmolyte pool to ensure retention of the optimal stability-flexibility balance of proteins. It will be interesting to determine if some of the other osmolytes that increase in concentration with depth, e.g., proline betaine and GPE, also have protein-stabilizing ability against pressure stress.

#### Nucleic acids

Again, as in the case of perturbation by temperature, stabilizing osmolytes may be critical for nucleic acids to maintain the appropriate levels of stability of higher order structures under high- or varying hydrostatic pressures. Certain of the higher order structures of nucleic acids are known to be pressure-sensitive. Whereas canonical DNA duplex structures like B-DNA are relatively resistant to change in pressure, non-canonical DNA structures such as DNA hairpins and G-quadruplexes have recently been shown to exhibit substantial sensitivities to pressure (e.g., Oliva et al. 2021). These effects could result in pressure-induced alteration of a number of critical processes that depend on higher order structures of DNA and RNA, e.g., initiation of translation, response to regulatory ligands, and control of splicing (Mortimer et al. 2014). Whereas the roles of intrinsic pressure-adaptive differences in nucleic acids remain to be investigated, studies of osmolytes’ capacities for offsetting perturbation of nucleic acid structures by elevated hydrostatic pressure have produced results that mirror those of studies of pressure-protein interactions.

In a study of a DNA hairpin, elevated pressures favored unfolding of the hairpin due to a negative volume change upon unfolding (Arns et al. 2019). When an osmolyte system designed around the cytosolic composition of a deep-sea shrimp was used (580 mmol/L TMAO, 100 mmol/L glycine, 35 mmol/L glycine betaine, and 20 mmol/L valine), pressure denaturation of the DNA hairpin was greatly reduced; this stabilizing effect was greater at 4 °C, a temperature near deep-sea temperatures, than at 20 °C, and appeared to be due principally to the effects of TMAO. In this same study, the effects of elevated pressure were examined on a G-quadraplex DNA structure. Once again, high pressures destabilized the DNA structure, but TMAO was capable of offsetting this perturbation, an effect that might be explained by unfavorable interactions between TMAO and the nucleobases and phosphate backbone. Thus, for nucleic acids as well as proteins, TMAO’s exclusion from the macromolecule’s surface affords stabilization of the folded state of the macromolecule. As predicted, urea had effects that were opposite those of TMAO. The folded structures were destabilized by urea and their sensitivity to pressure-induced unfolding increased.

In another study of pressure and osmolyte effects on higher order structure of DNA, a poly dA loop DNA hairpin was examined using a single-molecule Förster resonance energy transfer (FRET) procedure that allowed precise estimation of the amounts of the hairpin in each of the two states the system can occupy: closed and open (unfolded) (Patra et al. 2019). The important roles played by DNA hairpin structures, notably in regulation of gene expression, make hairpins a relevant study system for examination of stressor effects. The stabilization of hairpins depends on base stacking interactions, hydrogen bonding, and hydration state, all of which are sensitive to the solution bathing the DNA hairpin. The results of these FRET experiments cast important light on the role of the cellular solution in governing stability of nucleic acid higher order structures. In addition to examining effects of pressure and two organic osmolytes, TMAO and urea, the authors studied the effects of ionic strength on hairpin structure. Higher ionic strength stabilized the hairpin and reduced the effect of pressure on the equilibrium between the open and closed states; the volume change associated with unfolding (Δ*V*^o^) changed from approximately – 18 cm^3^/mol at low ionic strength to − 5.9 to − 5.2 cm^3^/mol at higher ionic strength. The sensitivity of the hairpin’s response to pressure on the ionic strength and ionic composition of the study medium provides a caveat to investigators of processes such as nucleic acid folding equilibria: biologically realistic solution conditions are of critical importance if the goal of the study is to reveal effects similar to those that occur in vivo. This point is further buttressed by the findings that stabilizing organic osmolytes (here TMAO) and molecular crowding effects stabilize the folded state. The occurrence of high TMAO concentrations in deep-living animals thus appears to have another beneficial effect for these species. The effect of the molecular crowding reagent (Ficoll) shows that the crowded nature of the intracellular environment also is likely to play an important role in governing stability of high order nucleic acid structures, a phenomenon discussed in a subsequent section of this review.

A study of pressure and osmolyte effects on RNA yielded results that essentially parallel those found in the studies of DNA mentioned above (Gao et al. 2017a). Pressure destabilized the hairpin structure of the RNA (a 4U RNA thermometer of *Salmonella*), as did elevated temperature. TMAO stabilized the folded RNA structure in the face of both abiotic stressors. As in the study of the DNA hairpin (Patra et al. 2019), the folding of the 4U RNA hairpin also exhibited a strong sensitivity to ionic strength (concentration of KCl) and its folded structure was destabilized by addition of urea.

#### Membranes

Imposition of elevated hydrostatic pressure on a membrane system can have several consequences. The degree of order in the lipids of the bilayer may be increased as a result of closer packing of the phospholipid acyl chains. Likewise, the transition between gel-like regions and fluid (liquid–crystalline) regions may be affected; pressure generally favors the gel phase over the liquid–crystalline phase due to volume differences between the two phases (Maiti and Daschakraborty 2022). Where phase discontinuities exist, the barrier function of a membrane may be compromised, leading to increased trans-membrane movement of solutes (Hazel 1995). Thus, perturbation by elevated pressure that leads to phase discontinuities can threaten one of the most critical functions of membranes. However, elevated pressure has been shown in some studies to reduce membrane permeability (see Maiti and Daschakraborty 2022).

In view of these complex effects of elevated pressure on the biophysical properties of membranes, it is of interest to see if the addition of a stabilizing osmolyte like TMAO enhances or counteracts pressure’s perturbing effects. In fact, TMAO has effects that resemble those of pressure in many ways (Maiti and Daschakraborty 2021, 2022; Manisegaran et al. 2019). Addition of TMAO to an in vitro model membrane system was found to increase the temperature of the gel-to-fluid phase transition and to increase the degree of order in the bilayer (Manisegaran et al. 2019). Lipid domains also coalesced in the presence of TMAO. Two recent molecular dynamics studies (Maiti and Daschakraborty 2021, 2022) yielded results consistent with the in vitro study and further extended the understanding of TMAO-membrane-pressure interactions. Thus, TMAO increased lipid packing density and shifted the fluid-to-gel transition pressure to a lower pressure, effects that may stem from dehydration of the lipid headgroups by TMAO. TMAO also reduced the lateral diffusion of lipids in the bilayer. Consistent with the increases in lipid acyl chain packing density, elevated pressure increased the thickness of the membrane. The addition of TMAO led to further increase in thickness, at least up to pressures of ~ 800 bar when the membrane as a whole entered a gel phase. A recent study of the effects of glycine betaine on model membranes showed that GB has effects similar to those of TMAO, albeit the strengths of the effects were generally lower (Shakhman and Harries 2021).

From the perspective of evaluating a potential adaptive role of TMAO and GB on membranes of deep-living species, it is easily seen that the effects of the elevated pressures and low temperatures of the deep-sea environment (imposition of a pressure of 1000 atm is equivalent to a reduction in temperature of 13–21 °C (Cossins and Macdonald 1989)) would be strengthened by high concentrations of TMAO and/or GB; all three factors would tend to reduce membrane fluidity and favor the fluid-to-gel transition. Suffice to say, although TMAO and GB have protective effects on proteins and nucleic acids under high pressure, accumulation of TMAO and GB in deep-living animals seems to be maladaptive in terms of the two osmolytes’ effects on membrane biophysical state.

This conclusion leads to the question of whether other organic osmolytes that exhibit depth-related accumulation patterns might help to offset the potential over-stabilization of membranes by TMAO and GB. Above, I pointed out how GPC and *scyllo*-inositol increased in concentration with depth of capture in deep-sea amphipods. Furthermore, other osmolytes increased as well, e.g., GPE and proline betaine (Downing et al. 2018). In the absence of evidence of how these four osmolytes affect the pressure responses of membranes, one can only speculate about the possibility that they might mitigate the stabilizing effects of high pressure, low temperature, and high [TMAO]. The uncertainty surrounding this issue certainly appears to warrant additional research on the pressure relationships of osmolytes on large molecular systems like membranes.

In light of the synergistic effects of low temperature, high pressure, and high TMAO/GB levels on membranes, it would seem that maintaining membrane function under deep-sea conditions would necessitate altering the lipid composition of the membrane to offset the stabilizing effects of the three deep-sea variables that reduce fluidity. In other words, *homeoviscous adaptation* of the bilayer structure may be key to offsetting these three order-enhancing factors (Ernst et al. 2016; Maiti and Daschakraborty 2022). There is in fact evidence that the membranes of deep-living organisms contain high levels of lipids that enhance fluidity and promote homeoviscous adaptation at depth (Cossins and MacDonald 1989; Gibbs and Somero 1990; Pond et al. 2014).

A more nuanced analysis that allowed separation of the effects of high pressure from those of low temperature has added an additional level of complexity to the lipid adaptation story (Winnikoff et al. 2021). Twenty-one species of ctenophores from different latitudes (temperatures from − 2 to 28 °C) and depths (surface to ~ 4000 m) were examined to search for patterns of lipid adaptation to these two physical stressors. For species with body temperatures less than ~ 7.5 °C, depth predicted the unsaturation levels of fatty acids; the deeper the species, the more fluid (unsaturated) were the lipids. In contrast, in species from the upper 200 m of the water column, temperature-related variation in fatty acid chain length was the dominant pattern in lipid adaptation. This study of ctenophores calls out for additional study of other taxa, to determine how temperature and pressure influence membrane composition and biophysical properties.

Lastly, the depth-related relationships in urea and TMAO concentrations in marine elasmobranchs merit attention (Fig. 5B). Urea tends to favor the fluid (liquid-crystalline) state of membranes, opposite to the effects of TMAO (e.g., Maiti and Daschakraborty 2022). Therefore, the co-occurrence of urea and TMAO in elasmobranchs may help to thwart loss of optimal liquid–crystalline states in deep-living members of this group of fishes. In fact, in vitro studies using model membranes show that this type of counteracting solute effect does apply to membranes as well as to proteins (Shakhman and Harries 2021).

## Need for holistic analysis: moving from the in vitro to the complexities of the in vivo

The foregoing discussion of the influences of osmolytes on the largest molecules and molecular assemblages of the cell needs to be expanded into a more holistic analysis, one which takes into account the summed effects of molecules of all size classes on macromolecular stability and function. This analysis includes the stabilizing effects macromolecules may have on themselves, an important issue not yet addressed in this review. Below, I introduce several phenomena that represent important areas that require much additional study in our efforts to understand the true in vivo properties of macromolecules. The following sections are not intended as critical reviews, but rather as suggestions for lines of study that may provide novel and important insights into the factors that govern stability of large molecular systems and thereby exert important influences on their functions.

### Molecular crowding and excluded volume effects

When one sums together the effects of all classes of molecules on the stability of the macromolecular machinery that underlies—indeed, defines—living processes, it is important to appreciate effects stemming from what is termed *molecular crowding* (Fulton 1982; Gao et al. 2016, 2017b; Minton 1981). Whereas most in vitro studies employ dilute solutions of the macromolecule(s) of interest, within cells the summed concentrations of proteins, nucleic acids, lipids, and carbohydrates may reach levels near ~ 400 mg/ml (Gao et al. 2017b). Together, these large molecules may constitute up to ~ 40% of the cellular volume. Thus, the expression *molecular crowding* is apt, as is the closely related concept of *excluded volume*, which recognizes the fact that macromolecules do not have the extent of aqueous volume surrounding them that they find in dilute solutions. There is a lesser volume of water for them to unfold into compared to the typical conditions found in studies done in vitro, and this reduced availability of water can greatly affect stability (Fields et al. 2001; Minton 1981).

The effects of crowding have received a great deal of study in efforts that attempt to simulate intracellular conditions. These investigations have shown that the various crowding agents used in vitro have different effects on macromolecular structure and function (Gao et al. 2016). Thus, depending on the crowding agent used, the in vitro results exhibit different degrees of agreement with in vivo studies. Many studies have shown that crowding can have strongly stabilizing effects on macromolecules. In the case of proteins, crowding agents such as Ficoll can stabilize enzymes against elevated temperatures and hydrostatic pressures (Gao et al. 2017b). The effects of temperature and pressure on nucleic acids also may depend on crowding conditions (Gao et al. 2016, 2017b). As Leamy et al. (2016) emphasize in their review of RNA higher order structures, differences in RNA structure and stability between in vitro and in vivo conditions are due in large measure to crowding effects. Because in vitro studies generally neglect crowding, the results of such experiments commonly lack “*direct cellular relevance*” (Leamy et al. 2016). The discrepancy between in vitro and in vivo conditions is clearly illustrated by studies of Tyrrell et al. (2013). They used a protocol (selective 2-hydroxyl acylation analyzed by primer extension (SHAPE)) in which a reagent that covalently modifies RNA structure and that penetrates into living cells is used. This approach thus allows comparisons of RNA stability under cellular conditions as well as in dilute experimental media. SHAPE labeling in the cells was much reduced compared to in vitro conditions, a finding that the authors suggest shows that the crowded intracellular solution exerts a strong stabilizing effect on RNA structure.

**Fig. 6:**
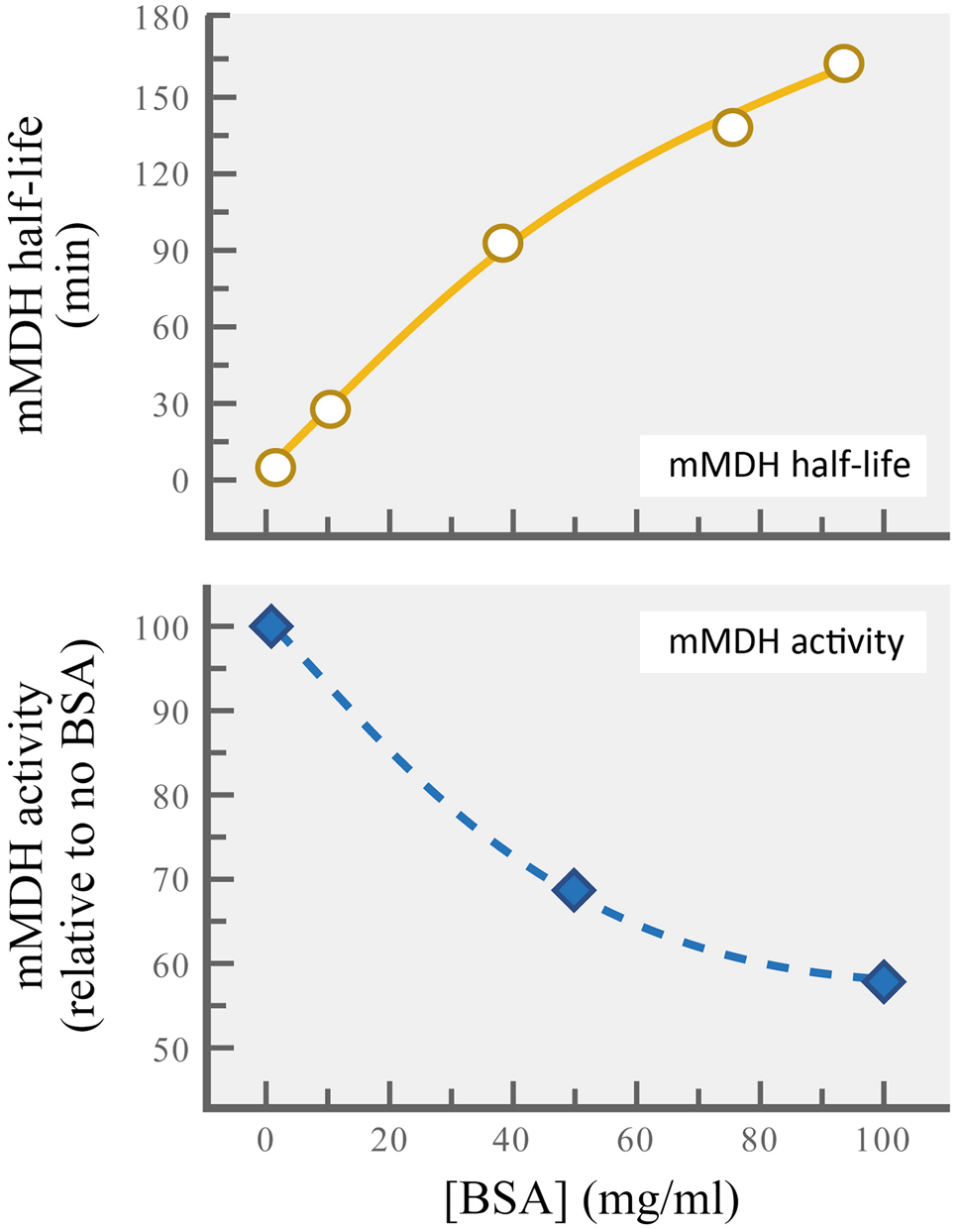
The effects of bovine serum albumin (BSA) concentration on the thermal stability (upper panel) and catalytic activity (lower panel) of the mitochondrial paralog of malate dehydrogenase (mMDH). (Figure redrawn after Lin et al. 2002)

As in the case of RNA, crowding is likely to affect protein stability which, in turn, may alter rates of protein function (Fields et al. 2001; Fulton 1982; Lin et al. 2002). An example of these types of interactions is provided by work of Lin and colleagues (Lin et al. 2002), who studied an exceptionally labile ortholog of malate dehydrogenase. The mitochondrial ortholog of MDH (mMDH) rapidly loses activity when present in dilute solutions, but its stability is likely to be much higher in the protein-rich cellular compartment in which it occurs, the mitochondrial matrix. The stability of mMDH (indexed by its half-life at 42 °C) shows a regular increase when increasing amounts of bovine serum albumin (BSA) are added to in vitro preparations of the enzyme to simulate effects of crowding (Fig. 6, upper panel). Concurrent with the increase in stability caused by BSA is a decrease in maximal activity (Fig. 6, lower panel). How are these data to be interpreted in the context of the intramitochondrial solution that cMDH occurs in within the mitochondrial matrix? The total protein concentration in the mitochondrial matrix is extremely high; estimates are that it may reach ~ 50% by mass (Srere 1981). These highly stabilizing crowded conditions would strongly enhance the rigidity of protein conformation. Thus, Lin et al. (2002) proposed that proteins that occur in such a crowded solution would benefit from being intrinsically labile in conformation, such that only under the native (crowded) condition of the matrix would they attain the appropriate balance between conformational stability and flexibility. Because the rate-governing step in the MDH reaction is a conformational change associated with ligand binding or release (Dong et al. 2018), the reduction in specific activity of mMDH with rising thermal stability can be rationalized. This example of mMDH suggests an important type of interplay between protein stability and solution chemistry: selection favors adjustment of protein stability not only to adapt the protein to temperature, but also to ensure that the appropriate balance between flexibility and rigidity is attained under the solution conditions (here, of extreme molecular crowding) within which the protein functions.

### Liquid–liquid phase separation

The foregoing discussion of mMDH and the working environment of an enzyme in the protein-rich mitochondrial matrix is a good introduction to a topic that has emerged as an exciting new area in cell biology: *liquid–liquid phase separation* (Alberti et al. 2019; Arns and Winter 2019). There is increasing recognition of and interest in the occurrence in cells of different liquid phases with distinct chemical compositions and different levels of molecular crowding. As the above discussion of molecular crowding would suggest, the existence of multiple liquid phases, which occur in the cytoplasm, the nucleus, and different organelles, has wide-reaching effects on the biology of cells. Most familiarly, these different liquid phases foster intracellular compartmentation, separating the biochemical systems that support different types of metabolic activity. In addition, in the different liquid phases within a cell, the stabilizing potentials of the cellular solution may differ widely, as discussed for the mitochondrial matrix.

The phenomenon of liquid–liquid phase separation is under intense study with a wide range of techniques that may reveal how the process is driven and what its consequences are for cellular organization and function. I will not further discuss this newly emerging area of research, other than to point out that the occurrence of multiple liquid phases with heterogeneous biochemistry adds another element that must be considered when attempting to link the in vitro to the in vivo and thereby interpret how the local solution environment of macromolecules influences their stability and function. It also seems fair to conjecture that proteins may evolve to have stabilities that fit them for function under the specific solution conditions of the liquid microenvironment in which they function. This is a new aspect of protein evolution that would seem to warrant study. Perhaps the occurrence of multiple paralogs of proteins that are distributed in different cell types or cellular compartments reflects in part the selection for intrinsic stabilities that fit each paralog for the specific stabilizing condition of the solution in which it functions.

### Might protein concentration—crowding effects—influence osmolyte accumulation patterns?

Much as the level of molecular crowding may influence selection for intrinsic protein stability, the concentration of protein in a tissue could, in principle, affect osmolyte system composition and concentration. Tissues with low protein content, for example, would provide relatively little protein stabilization due to crowding effects. In such tissues, accumulation of protein-stabilizing osmolytes under stress from temperature or pressure might be especially important. Furthermore, the type of stabilizing osmolyte that is selected for accumulation might vary between protein-rich and protein-poor tissues. To my knowledge, this question has not been studied to date.

Further study of depth-related patterns of tissue biochemistry offers a possible approach to elucidate this potential relationship between osmolyte composition and stabilization due to crowding. In pelagic marine teleosts the concentrations of protein and levels of enzyme activity in white locomotory muscle decrease with increasing depth of distribution (Childress and Somero 1979). This trend seems absent in brain and heart, so the possibility arises that osmolyte accumulation patterns with depth might show tissue-specific patterns related to differences among tissues in stabilizing potential due to crowding effects. Because tissue osmolality would be expected to be the same in all tissues, variation in osmolyte (piezolyte) composition would involve choice of type of osmolyte, not overall osmolyte concentration. As pointed out in the context of acclimatization of osmolyte systems under thermal stress, the potential influences of the type of tissue that is examined must be taken into account in studies of osmolyte accumulation patterns.

### Viscosity of cellular solutions: effects on structure and function of macromolecules

Another phenomenon closely related to molecular crowding is the *viscosity* of the cellular solution. In general, the higher the concentrations of dissolved solutes—large and small—the greater is the viscosity of a solution. Furthermore, solutes differ in their effects on water’s viscosity, so overall osmolality is not sufficient to predict solution viscosity (Diamant et al. 2001). Viscosity has wide-ranging effects on macromolecular function and structure. One well-known effect of viscosity is its influence on the rates of diffusion of molecules of all sizes. Thus, when viscosity is high, rates of enzymatic activity may be reduced due to less frequent contact of substrates with their binding sites on enzymes. Increases in viscosity also may enhance macromolecular stability and thereby influence rates of function (Beece et al. 1980). Thus, the decreased conformational flexibility of protein structure at elevated solution viscosities can have inhibitory effects on rates of biochemical processes through increasing energy barriers to conformational changes that are required for function.

Organic osmolytes differ in their effects on solution viscosity (Diamant et al. 2001), and these viscosity-based effects may help to account for the different stabilizing efficacies of osmolytes. In a comparison of four commonly occurring organic osmolytes—trehalose, glycine betaine, glycerol, and proline—it was discovered that all four osmolytes conferred similar stability on a protein (MDH) when the solutions had a common viscosity, even though the osmolytes differed in stabilizing efficacy when compared at a common concentration. Further study of viscosity-macromolecular stability relationships is warranted, including studies that examine how temperature effects on solution viscosity affect macromolecular structure and function and how stabilization by osmolytes—and the requirements for such stabilization—are influenced by temperature–viscosity relationships (see Somero et al. 2017).

In summary, to understand the complex interplay between the osmolyte systems of cells and macromolecular stability and function, a holistic analysis that examines all types of molecular interactions is required. For example, much as osmolytes influence macromolecular stability, so do the concentrations of the macromolecules themselves affect this stability through the ‘crowdedness’ they create in cellular fluids. The state of crowding in a cell—or, better, in a specific liquid phase compartment within the cell—may affect selection for macromolecular stability, because of the need to maintain the correct level of conformational flexibility for optimal macromolecular function. Macromolecular stability and activity are also highly influenced by the viscosity of the cellular solution. All size classes of solutes influence the viscosity of cellular solutions, often in solute-specific fashion. Understanding these complex interactions among macromolecules and osmolytes is important for elucidating processes of biochemical evolution and for designing experiments that attempt to generate data that reflect the in vivo characteristics of the molecules under study, a goal that should of course be central in studies of biochemistry.

## Lessons and opportunities for bio-technology

The strategies used by marine organisms to cope with physical and chemical stressors have important lessons to teach biotechnologists. In particular, the capacities of small organic solutes to favorably affect the structural and functional properties of macromolecules and large molecular assemblages like membranes offer useful pointers for investigators attempting to enhance the stabilities and longevities of the biological material they study. With increased interest in using RNAs in numerous biotechnological and biomedical contexts, the relatively recently discovered effects of osmolytes on higher order RNA structures may have especially important lessons to teach. Likewise, through understanding the complexities of biological solutions, investigators may be better able to design artificial media that closely mimic actual cellular conditions and, thereby, are likely to facilitate the discovery of the true in vivo properties of the study system. For a variety of industrial purposes, stabilizing osmolytes, especially the so-called *extremolytes* found in microbes tolerant of extreme temperatures and salinities, are viewed as an “unexploited goldmine” of potentially valuable chemicals (Becker and Wittmann 2020). Exploitation of these and other stabilizing osmolytes may demand improved means for high-scale production of natural products and the development of new, even more powerful stabilizers.

Studies of naturally occurring osmolytes may offer guidance into the fabrication of novel organic molecules with favorable effects on macromolecules. Assemblage of two or more stabilizing osmolytes into a single molecule may be a promising biosynthetic strategy for fabricating especially powerful stabilizers. Glycerol-phosphoryl-choline offers an example of how three individual stabilizers can be combined into a single, extremely potent stabilizer. Such an approach has been tried using different natural stabilizers as starting points for creating novel osmolytes. TMAO-based efforts have utilized two approaches. In one, novel molecules with two trimethylammonium groups were synthesized (Levy-Sakin et al. 2014). Although these new compounds were stabilizing of proteins, they were less effective than TMAO itself. Interestingly, the stabilizing potential of the “twin-TMAO” solutes was influenced by the number of carbons separating the trimethylammonium groups. The addition of one more carbon between the groups resulted in higher stabilizing potential than was found in the “twin TMAO” that lacked a separating carbon. A second approach using TMAO as a tool in stabilizer design involved synthesis of a vinyl polymer with a TMAO side-chain (Anjum et al. 2021). This novel compound had excellent protein-stabilizing ability, which supports the utility of such synthetic approaches using known stabilizers like TMAO as guides for producing new types of macromolecular stabilizers.

The potent anionic glycoside stabilizers found in hyperthermophilic microbes (Fig. 3) also have served as guides for designing novel stabilizers (Faria et al. 2008). These investigators used mannosylglycerate (MG) as a starting point and produced a number of synthetic anionic osmolytes that had varying efficacies in preventing thermal inactivation of several different proteins. Some of the de novo synthesized osmolytes were less effective than MG, whereas others were superior, at least in some contexts. For example, the synthesized compound mannosyl-lactate (ML) was superior to the naturally occurring stabilizer MG in protecting Staphylococcal nuclease (SNase; Fig. 3); ML and MG were equally effective in protecting malate dehydrogenase. The stabilizing effects of the naturally occurring and synthesized anionic osmolytes generally were similar to the effects of the most strongly stabilizing ions of the Hofmeister Series, phosphate and sulfate. Additional studies of these sorts are warranted to further explore the lessons that naturally occurring macromolecular stabilizers may have to teach us about creating new types of stabilizers that have potential for exploitation in biotechnology, notably in developing strategies for long-term stabilization and preservation of biological materials.

Looking more broadly at research on marine natural products that aims to characterize the rich variety of small organic compounds in marine organisms, the so-called secondary metabolites (for review, see Liu et al. 2019), it would seem that studies that focus on marine natural products’ diverse functions as anti-fouling agents, predatory deterrents, anti-viral agents, anti-cancer drugs and settlement cues might be broadened in focus to include a search for macromolecular stabilizers. The chemical structures of these secondary metabolites generally seem unlikely to confer upon them stabilizing potential and their generally low concentrations in cells argue against an important role in osmolyte systems. Nonetheless, remaining alert for the possibility of macromolecule stabilizing functions for secondary metabolites seems appropriate. Perhaps there exist secondary metabolites that protect structures of specific types of macromolecules through direct binding to them, a stabilization mechanism that differs from the water structure mediated effects characteristic of stabilization by other stabilizing osmolytes.

## Summary and conclusions

- In most marine organisms, small organic molecules (organic osmolytes) rather than inorganic ions account for the largest share of intracellular osmolality. In addition to their roles in establishing osmotic balance, osmolytes play important roles in offsetting perturbation of large molecular systems (proteins, nucleic acids and membranes) by extremes of temperature, hydrostatic pressure, and salinity. A key function of these osmolyte adaptations is maintenance (or restoration) of an optimal balance between macromolecular rigidity and flexibility, a balance that is key to optimal function.
- A given type of osmolyte typically has similar stabilizing (e.g., TMAO) or destabilizing (e.g., urea) effects on all types of large molecular systems. Adaptive alterations in osmolyte composition and concentration thus may have a global influence on the stabilities of macromolecules and membranes and serve important roles in establishing environmental optima and tolerance ranges across all biochemical systems.
- Stabilizing osmolytes differ widely in how effectively they enhance macromolecular stability. Thus, organisms can assemble diverse types of cellular osmolyte solutions in accord with the magnitude of abiotic stress that is impacting the stability-flexibility balance of proteins, nucleic acids, and membranes. Extremophiles provide compelling evidence for this type of adaptive response: high temperatures and high hydrostatic pressures favor the accumulation of especially stabilizing osmolyte mixtures, relative to those characteristic of mesophilic organisms.
- The abilities to finely tune the stabilizing potential of osmolyte systems may offer organisms an important tool for adjusting thermal optima and tolerance limits in the face of changes in body temperatures that occur over different time-courses. Relatively rapid responses may occur on a diurnal basis, e.g., during the tidal cycle in intertidal species, and more prolonged changes may occur in response to seasonal temperature change. The potential importance of acclimatization effects in counteracting stress from global warming suggests a role for osmolyte systems over multi-generational periods as well. Warming may occur too rapidly for the intrinsic thermal responses of macromolecules to evolve adequately, so reliance on extrinsic protection by modulation of osmolyte systems may be crucial for survival.

## Data Availability

All data generated and analyzed during this study are included in this published article.
